# Association Between Long‑Term Exposure to Air Pollution and the Rate of Mortality After Hip Fracture Surgery in Patients Older Than 60 Years: Nationwide Cohort Study in Taiwan

**DOI:** 10.2196/46591

**Published:** 2024-03-18

**Authors:** Shu-Han Chuang, Yi-Jie Kuo, Shu-Wei Huang, Han-Wei Zhang, Hsiao-Ching Peng, Yu-Pin Chen

**Affiliations:** 1 Division of General Practice Department of Medical Education Changhua Christian Hospital Changhua Taiwan; 2 Department of Orthopedics Wan Fang Hospital Taipei Medical University Taipei City Taiwan; 3 Department of Orthopedics School of Medicine, College of Medicine Taipei Medical University Taipei Taiwan; 4 Department of Applied Science National Taitung University Taitung City, Taitung County Taiwan; 5 MetaTrial Research Center Biomedica Corporation New Taipei Taiwan; 6 Program for Aging China Medical University Taichung Taiwan; 7 Institute of Population Health Sciences National Health Research Institutes Miaoli Taiwan; 8 Department of Electrical and Computer Engineering Institute of Electrical Control Engineering National Yang Ming Chiao Tung University Hsinchu Taiwan

**Keywords:** air pollution, hip fracture, mortality, exposure, older adult, environmental hazard, hazard, morbidity

## Abstract

**Background:**

To enhance postoperative patient survival, particularly in older adults, understanding the predictors of mortality following hip fracture becomes paramount. Air pollution, a prominent global environmental issue, has been linked to heightened morbidity and mortality across a spectrum of diseases. Nevertheless, the precise impact of air pollution on hip fracture outcomes remains elusive.

**Objective:**

This retrospective study aims to comprehensively investigate the profound influence of a decade-long exposure to 12 diverse air pollutants on the risk of post–hip fracture mortality among older Taiwanese patients (older than 60 years). We hypothesized that enduring long-term exposure to air pollution would significantly elevate the 1-year mortality rate following hip fracture surgery.

**Methods:**

From Taiwan’s National Health Insurance Research Database, we obtained the data of patients who underwent hip fracture surgery between July 1, 2003, and December 31, 2013. Using patients’ insurance registration data, we estimated their cumulative exposure levels to sulfur dioxide (SO_2_), carbon dioxide (CO_2_), carbon monoxide (CO), ozone (O_3_), particulate matter having a size of <10 μm (PM_10_), particulate matter having a size of <2.5 μm (PM_2.5_), nitrogen oxides (NO_X_), nitrogen monoxide (NO), nitrogen dioxide (NO_2_), total hydrocarbons (THC), nonmethane hydrocarbons (NMHC), and methane (CH_4_). We quantified the dose-response relationship between these air pollutants and the risk of mortality by calculating hazard ratios associated with a 1 SD increase in exposure levels over a decade.

**Results:**

Long-term exposure to SO_2_, CO, PM_10_, PM_2.5_, NO_X_, NO, NO_2_, THC, NMHC, and CH_4_ demonstrated significant associations with heightened all-cause mortality risk within 1 year post hip fracture surgery among older adults. For older adults, each 1 SD increment in the average exposure levels of SO_2_, CO, PM_10_, PM_2.5_, NO_X_, NO, NO_2_, THC, NMHC, and CH_4_ corresponded to a substantial escalation in mortality risk, with increments of 14%, 49%, 18%, 12%, 41%, 33%, 38%, 20%, 9%, and 26%, respectively. We further noted a 35% reduction in the hazard ratio for O_3_ exposure suggesting a potential protective effect, along with a trend of potentially protective effects of CO_2_.

**Conclusions:**

This comprehensive nationwide retrospective study, grounded in a population-based approach, demonstrated that long-term exposure to specific air pollutants significantly increased the risk of all-cause mortality within 1 year after hip fracture surgery in older Taiwanese adults. A reduction in the levels of SO_2_, CO, PM_10_, PM_2.5_, NO_X_, NO, NO_2_, THC, NMHC, and CH_4_ may reduce the risk of mortality after hip fracture surgery. This study provides robust evidence and highlights the substantial impact of air pollution on the outcomes of hip fractures.

## Introduction

Hip fracture, a major concern for older adults, is associated with high mortality and morbidity as well as a high economic burden on the health care system [1,2]. Hip fracture surgery often leads to poor functional outcomes, such as reduced quality of life, substantial care demands, and limited mobility [3-9]. Several studies have reported an increased rate of mortality (12% to 40%) within 1 year after hip fracture in older adults [10-15]. Pneumonia and cardiovascular diseases are the predominant causes of mortality after hip fracture in these individuals [16-19]. Perioperative factors associated with the risk of 1-year mortality after hip fracture surgery include high baseline Charlson Comorbidity Index (CCI) scores, high American Society of Anesthesiologists grades, and prolonged surgery delay [4,20-22]. To adopt stratified care approaches, such as prioritizing geriatric patients with hip fractures at high risk of mortality for intensive care and to reduce the rate of mortality, clinicians must be aware of the relevant prognostic factors [15].

Since the 1990s, air pollution has been recognized as a major global environmental problem, which leads to increased mortality and morbidity rates [23-26]. Exposure to air pollution is associated with the risk of mortality from various conditions, such as cancer [27], critical illness [28], chronic obstructive pulmonary disease [29], COVID-19 [30], and major surgery [31-33]. However, few studies have investigated the effects of air pollution on the outcomes of hip fractures.

A recent meta-analysis revealed that high risks of osteoporosis and hip fracture were positively associated with increased exposure to air pollutants [34]. However, most studies on air pollution have focused only on bone marrow density and hip fracture incidence, but not on further prognosis or outcome. In 2022, Shi et al [35] reported that exposure to particulate matter having a size of <2.5 μm (PM_2.5_), particulate matter having a size of <10 μm (PM_10_), and nitrogen dioxide (NO_2_) may increase the risk of mortality within 30 days after hip fracture. Despite the limited samples of air pollutants collected and the lack of long-term evaluation, the aforementioned study may be the only one on the effects of air pollution on mortality after hip fracture surgery.

Emerging evidence underscores the need for a more detailed understanding of the relationship between air pollution and post–hip fracture mortality, particularly given the complex interplay of factors involved in the health outcomes of older adults. Beyond the direct respiratory effects, air pollution has been implicated in systemic inflammation [36-42] and oxidative stress [43-52], both of which are central to the pathophysiological processes underlying hip fracture complications and mortality [53,54]. In other words, this complex interplay might go beyond immediate respiratory implications and include the broader landscape of systemic health. Besides, the older population, particularly those with preexisting comorbidities, may be disproportionately vulnerable to the adverse effects of air pollution due to reduced physiological reserves and impaired reparative mechanisms [55]. It is essential to recognize the holistic impact of air pollution, not only as a respiratory hazard but as a systemic disruptor influencing the delicate balance of physiological processes in older adults.

Furthermore, hip fracture recovery is a complex process that involves various mechanisms that can be influenced by systemic factors. Investigations into the specific impact of long-term exposure to a comprehensive array of air pollutants on the 1-year postoperative mortality risk in this population have been limited, necessitating a deeper exploration into the biological underpinnings. As we consider the theoretical framework of hip fracture pathogenesis, we must delve into the ways air pollution may intricately contribute to the vulnerability and subsequent mortality risk in older adults following hip fracture surgery. By expanding our focus beyond the immediate postoperative period, we can unravel the enduring effects of air pollution on recovery trajectories and long-term outcomes in this vulnerable population.

Therefore, we conducted this retrospective study to investigate the effects of decade-long exposure to various air pollutants on the risk of mortality after hip fracture surgery in older Taiwanese patients. We hypothesized that long-term exposure to air pollution would have an impact on the rate of 1-year mortality after hip fracture surgery. This study investigated the effects of decade-long exposure to 12 air pollutants—sulfur dioxide (SO_2_), carbon dioxide (CO_2_), carbon monoxide (CO), ozone (O_3_), PM_10_, PM_2.5_, nitrogen oxides (NO_X_), nitrogen monoxide (NO), NO_2_, total hydrocarbons (THC), nonmethane hydrocarbons (NMHC), and methane (CH_4_)—on older Taiwanese adults who underwent hip fracture surgery. This study seeks to address these critical knowledge gaps by using a population-based approach and comprehensive air pollutant exposure data, shedding light on the intricate relationship between air pollution and post–hip fracture outcomes. This knowledge has the potential to offer evidence that may serve as a foundation for government authorities to develop strategies aimed at mitigating air pollution. Such strategies could significantly alleviate the strain on the health care system.

## Methods

### Data Source

In this study, we collected relevant data from Taiwan’s National Health Insurance Research Database (NHIRD), which was launched by the Taiwanese government in 1995. This database contains the comprehensive medical information of approximately 98.29% of the population of Taiwan (approximately 23 million insured individuals) [56]; these data include the patient’s sex, date of birth, employment status, inpatient and outpatient diagnoses, medical procedures, drug use, treatment duration, and medical costs [57]. We obtained the patients’ baseline information from the Longitudinal Health Insurance Database 2000, a subset of the NHIRD, containing the data of 1 million randomly selected patients.

All data generated or analyzed during this study are included in this published paper and its Multimedia Appendix files. More detailed data sets are not publicly available due to restrictions set by the Taiwan Ministry of Health and Welfare regarding the NHIRD in Taiwan. Researchers interested in accessing the data from the NHIRD must obtain approval from the Health and Welfare. For further information on data availability and access, please refer to the “Data Availability” section.

### Study Population

Only patients with complete available data were included in this study. Patients with missing, inconsistent, or unknown records of baseline information, such as sex and birth year, were excluded from this study. The cohort included patients who underwent hip fracture surgery between January 1, 2000, and December 31, 2012. Additional exclusion criteria were as follows: unreasonable age (0 years old); younger than 60 years at baseline; with the outcome diagnosis prior to the start of the study (to prevent reverse causation bias); history of pathological fracture, open fracture, or major traffic accident before the initiation of the study; whose follow-up start date was the same as the follow-up end date. At last, participants were excluded if their survival date was before July 01, 2003, to ensure that the study population had a minimum of 10 years of exposure to air pollution. This criterion was established because the Environmental Protection Administration (EPA) data for this study was only available starting from July 1, 1993. Therefore, if a participant’s follow-up ended before July 1, 2003, their exposure to air pollution would be less than 10 years. The *ICD-9* (*International Classification of Diseases, Ninth Revision*) codes established in the inclusion and exclusion criteria were listed in Multimedia Appendix 1.

### Exposure Data Collection

We obtained information on the cumulative daily average levels of the aforementioned 12 air pollutants from 76 monitoring stations maintained by the Taiwan EPA, Executive Yuan. Data were collected for the period between July 1, 1993, and December 31, 2013. The cumulative daily average level of each pollutant was calculated over the 10 years before the end of follow-up. Then, the data were integrated with the patients’ residential postal codes obtained from their insurance registration data. Any change in residence during the assessment period was also considered. Specifically, changes in a patient’s place of residence during the study period were tracked by their information with insurance registration records, ensuring that any shifts in location were accounted for in the study’s data analysis to maintain accuracy and reliability.

### Outcomes and Confounders

All-cause mortality was the major outcome of interest. We followed up on each patient until we reached the primary end point, which was determined by 2 conditions: death (withdrawal from the National Health Insurance program) or end of the study period (December 31, 2013). The survival duration was calculated in months. Several confounders were identified and adjusted for, such as age, urbanization level, insurance amount, CCI score, hip fracture procedure, comedications, antiosteoporosis medication, ambient temperature, season, and lag 0-1. Patient data such as age, insurance amount, CCI score, hip fracture procedure, comedications, and antiosteoporosis medication were also obtained from the NHIRD database. Specifically, the insurance amount was evaluated as an average value for the assessment period of air pollution exposure. Comorbidities were defined as those occurring before the survival date. *ICD-9* codes or Anatomical Therapeutic Chemical Classification codes for the definition of hip fracture procedure, comedications, and antiosteoporosis medication were detailed in Multimedia Appendix 1. Information on the meteorological factor of ambient temperature was collected from the EPA. Data on the urbanization level were recorded according to the patients’ residence at the beginning of the follow-up period in accordance with the classification proposed by Liu et al [58]. Furthermore, the season was defined on the basis of the date. We also included the 2-day moving average of current- and previous-day levels of air pollutants before the primary end point (lag 0-1) as a confounder, considering the largest effect estimate reported in the literature [59-61].

### Statistical Analysis

To identify the patient characteristics associated with air pollution, the cohort was divided into 3 tertiles on the basis of the level of exposure to each pollutant. The tertiles were compared using the chi-square test or 1-way ANOVA. Post hoc tests were performed to estimate between-tertile differences if significance was indicated. We plotted crude cumulative incidence curves of mortality within 1 year after hip fracture surgery for the 3 tertiles; between-tertile differences were assessed through log-rank tests. Hazard ratios (HRs) for exposure at 1 SD increment for 10 years were calculated using Cox regression models to evaluate the dose-response effects between air pollutants and mortality risk. The regression models were adjusted for the aforementioned confounders. All tests were 2-sided; statistical significance was set at *P*<.05. All analyses were performed using the MetaTrial Research Platform (Biomedica Corp).

### Ethical Considerations

The Research Ethics Committee of Taipei Medical University, Taiwan, approved this study (TMU-JIRB N202203088). Because of the anonymity of patient data in the NHIRD, the requirement of informed consent was waived.

## Results

### Study Population

**Figure 1:**
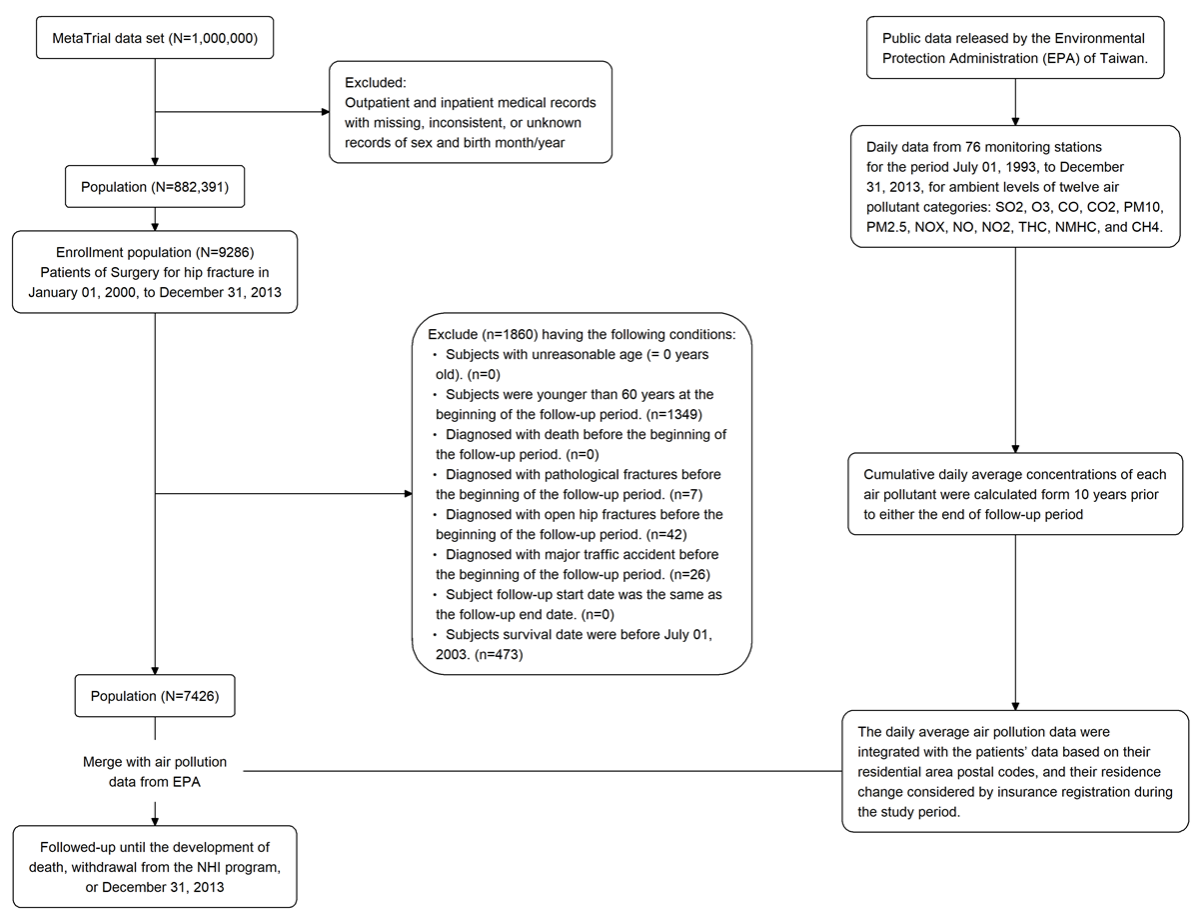
Flowchart for patient selection. NHI: National Health Insurance. CH_4_: methane; CO: carbon monoxide; CO_2_: carbon dioxide; NMHC: nonmethane hydrocarbons; NO: nitrogen monoxide; NO_2_: nitrogen dioxide; NO_X_: nitrogen oxides; O_3_: ozone; PM_10_: particulate matters having a size of <10 μm; PM_2.5_: particulate matters having a size of <2.5 μm; SO_2_: sulfur dioxide; THC: total hydrocarbons.

After the inclusion of patients with complete available data (n=882,391), those who underwent hip fracture surgery during the study period were identified (n=9286). Patients who met the exclusion criteria (1349, 75, and 473 for the aforementioned 3 criteria, respectively) were further excluded from the analysis. Finally, 7426 patients were included in the analysis. Figure 1 illustrates the selection process.

### Characteristics and Descriptive Results

Table 1 summarizes the characteristics of the included cohort (n=7426). The incidence of mortality was 929 (12.51%). The average age of the cohort was 78.54 years, and 2926 (39.40%) patients were men. The characteristics of patients exposed to each pollutant were assessed by dividing the cohort into 3 tertiles (Multimedia Appendices 2-13). Table 2 presents the mean and distribution values of each pollutant over the 10-year exposure period.

**Table 1:** Baseline characteristics of the study cohort.

Characteristics		Values
Deaths, n (%)		929 (12.51)
Men, n (%)		2926 (39.40)
**Age (years), n (%)**		
	60-79	3866 (52.06)
	≥80	3560 (47.94)
Age (years); mean (SD)		78.54 (8.07)
**Urbanization level, n (%)**		
	1 (highest)	3272 (44.06)
	2	2765 (37.23)
	3	711 (9.57)
	4 (lowest)	112 (1.51)
	Unknown	566 (7.62)
**Insurance amount (US $), n (%)**		
	Financially dependent	24 (0.32)
	0.032 to 631.23	3537 (47.63)
	631.26 to 1262.50	2373 (31.96)
	≥1262.53	119 (1.60)
	Unknown	1373 (18.49)
CCI^a^ score; mean (SD)		4.57 (2.97)
**Hip fracture procedure, n (%)**		
	Closed reduction of fracture with internal fixation	448 (6.03)
	Open reduction of fracture with internal fixation	3957 (53.29)
	Partial hip replacement	3021 (40.68)
Comedications, n (%)		6344 (85.43)
**Antiosteoporosis medication, n (%)**		
	Alendronate	752 (10.13)
	Risedronate	0 (0)
	Ibandronate	11 (0.15)
	Zoledronic	0 (0)
	Denosumab	0 (0)
	Raloxifene	236 (3.18)

^a^CCI score: Charlson Comorbidity Index.

**Table 2:** Mean and distribution of air pollutants over the exposure period.

	SO_2_^a^ (ppb^b^)	CO_2_^c^ (ppm^d^)	CO^e^ (ppm)	O_3_^f^ (ppb)	PM_10_^g^ (μg/m^3^)	PM_2.5_^h^ (μg/m^3^)	NO_X_^i^ (ppb)	NO^j^ (ppb)	NO_2_^k^ (ppb)	THC^l^ (ppm)	NMHC^m^ (ppm)	CH_4_^n^ (ppm)
Mean (SD)	4.14 (1.35)	398.46 (11.50)	0.55 (0.14)	28.08 (2.56)	55.68 (9.78)	33.7 (6.85)	25.43 (8.16)	7.41 (4.41)	18.02 (4.16)	2.31 (0.17)	0.30 (0.11)	2.01 (0.11)
T_1_^o^	3.59	393.97	0.47	27.31	50.25	29.98	20.59	4.69	15.87	2.22	0.25	1.96
T_2_^p^	4.06	401.51	0.61	28.83	58.45	36.49	28.32	7.76	20.29	2.36	0.32	2.05

^a^SO_2_: sulfur dioxide.

^b^ppb: parts per billion.

^c^CO_2_: carbon dioxide.

^d^ppm: parts per million.

^e^CO: carbon monoxide.

^f^O_3_: ozone.

^g^PM_10_: particulate matter <10 μm in size.

^h^PM_2.5_: particulate matter <2.5 μm in size.

^i^NO_X_: nitrogen oxides.

^j^NO: nitrogen monoxide.

^k^NO_2_: nitrogen dioxide.

^l^THC: total hydrocarbons.

^m^NMHC: nonmethane hydrocarbons.

^n^CH_4_: methane.

^o^T_1_: 33.33rd percentile.

^p^T_2_: 66.66th percentile.

### Cumulative Mortality Incidence

Table 3 presents the incidence of mortality within 1 year after hip fracture surgery across the tertiles, as well as the corresponding *P* value (1-way analysis of variance and post hoc tests). The cumulative incidence curves revealed substantial differences in the direction and strength of the associations between each air pollutant and the incidence of mortality (Figures 2-13).

**Table 3:** Incidence of mortality within 1 year after hip fracture surgery across tertiles.

Pollutants	Tertiles of average daily exposure, n/n (%)			*P* values	Total, n/n (%)
	T1 (lowest)	T2	T3 (highest)		
SO_2_^a^	229/2391 (9.58)	264/2541 (10.39)	436/2494 (17.48)	<.001	929/7426 (12.51)
CO_2_^b^	252/1271 (19.83)	86/1269 (6.78)	64/1272 (5.03)	<.001	402/3812 (10.55)
CO^c^	180/2438 (7.38)	300/2512 (11.94)	449/2476 (18.13)	<.001	929/7426 (12.51)
O_3_^d^	574/2343 (24.50)	178/2607 (6.83)	177/2476 (7.15)	<.001	929/7426 (12.51)
PM_10_^e^	274/2475 (11.07)	266/2335 (11.39)	389/2616 (14.87)	<.001	929/7426 (12.51)
PM_2.5_^f^	240/2417 (9.93)	245/2417 (10.14)	361/2418 (14.93)	<.001	846/7252 (11.67)
NO_X_^g^	199/2439 (8.16)	329/2511 (13.10)	401/2476 (16.20)	<.001	929/7426 (12.51)
NO^h^	215/2409 (8.92)	325/2541 (12.79)	389/2476 (15.71)	<.001	929/7426 (12.51)
NO_2_^i^	197/2475 (7.96)	311/2475 (12.57)	421/2476 (17.00)	<.001	929/7426 (12.51)
THC^j^	118/2442 (4.83)	231/2441 (9.46)	573/2442 (23.46)	<.001	922/7325 (12.59)
NMHC^k^	215/2442 (8.80)	305/2441 (12.49)	402/2442 (16.46)	<.001	922/7325 (12.59)
CH_4_^l^	100/2442 (4.10)	229/2441 (9.38)	593/2442 (24.28)	<.001	922/7325 (12.59)

^a^SO_2_: sulfur dioxide.

^b^CO_2_: carbon dioxide.

^c^CO: carbon monoxide.

^d^O_3_: ozone.

^e^PM_10_: particulate matters having a size of <10 μm.

^f^PM_2.5_: particulate matters having a size of <2.5 μm.

^g^NO_X_: nitrogen oxides.

^h^NO: nitrogen monoxide.

^i^NO_2_: nitrogen dioxide.

^j^THC: total hydrocarbons.

^k^NMHC: nonmethane hydrocarbons.

^l^CH_4_: methane.

**Figure 2:**
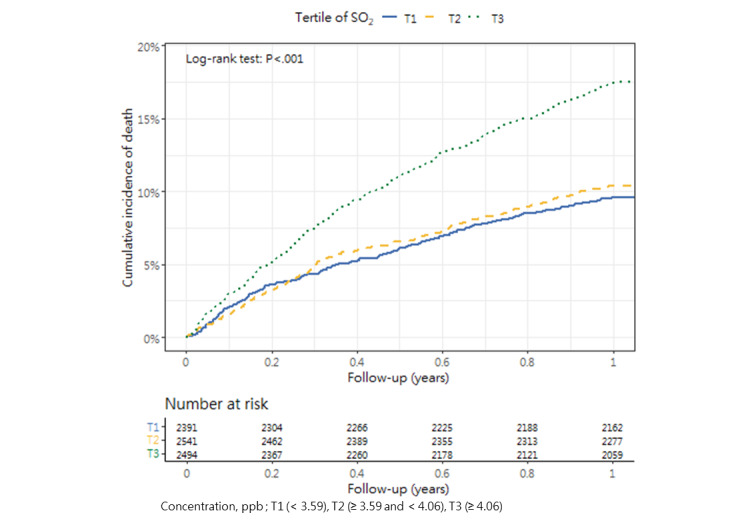
Cumulative incidence curves of mortality across tertiles of sulfur dioxide.

**Figure 3:**
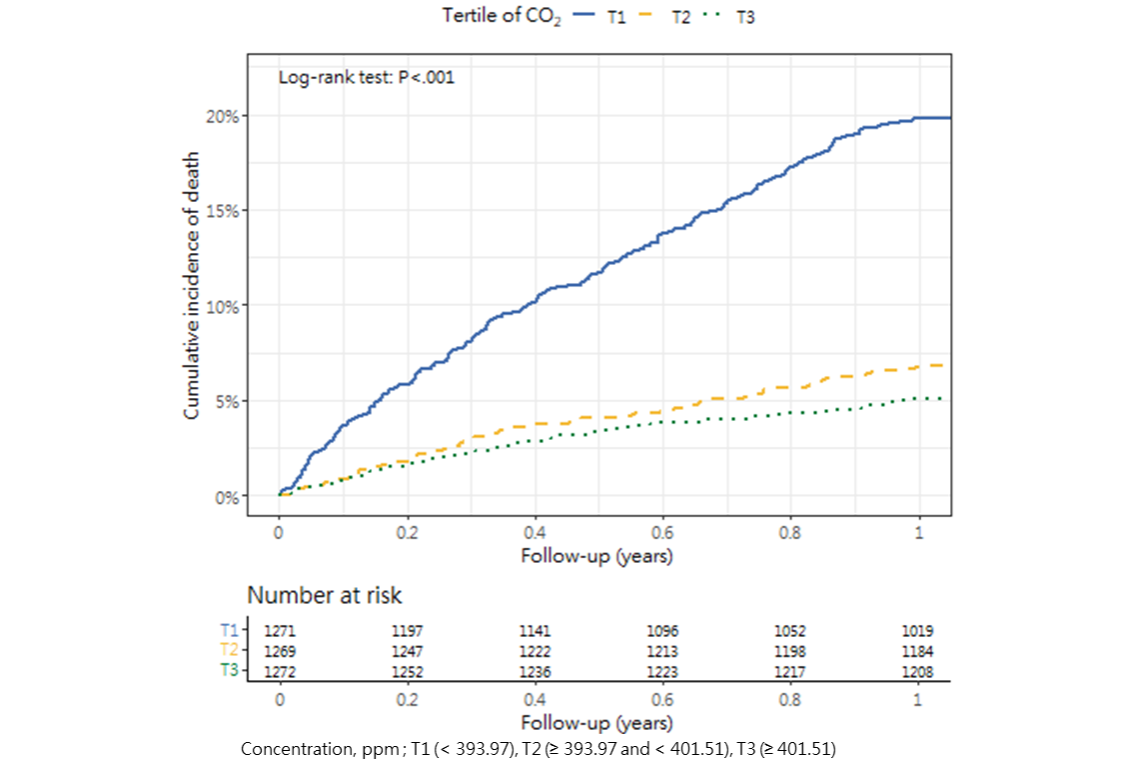
Cumulative incidence curves of mortality across tertiles of carbon dioxide.

**Figure 4:**
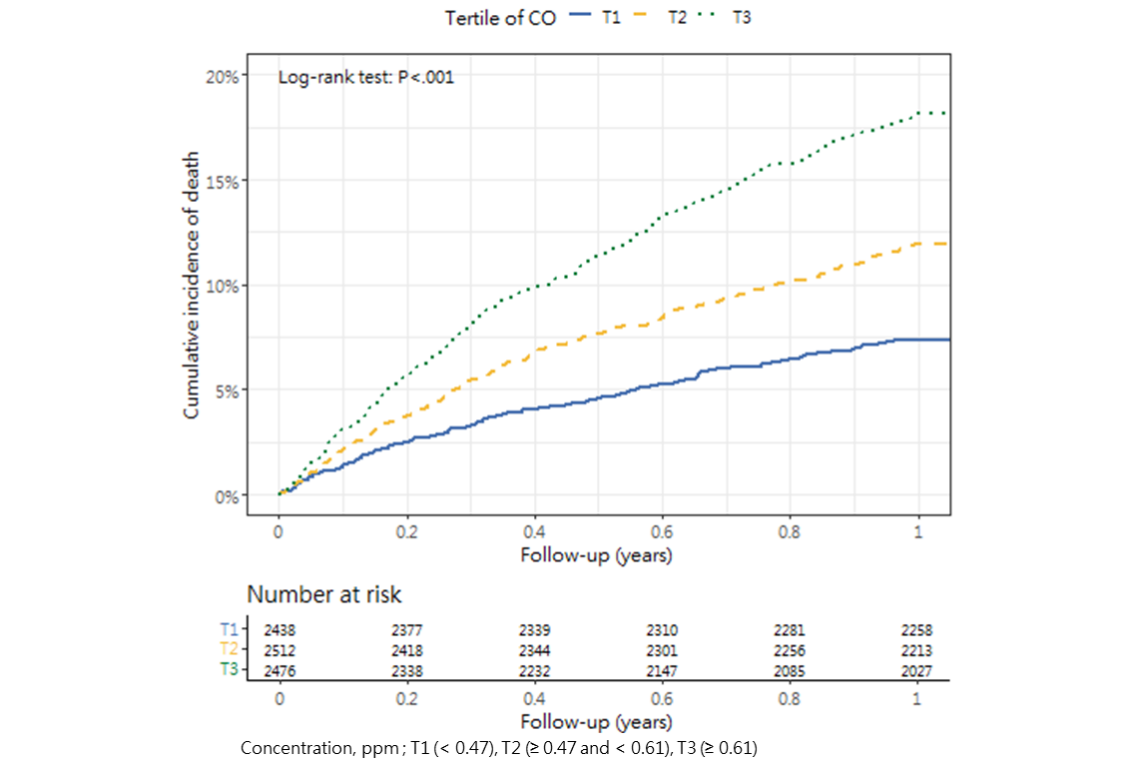
Cumulative incidence curves of mortality across tertiles of carbon monoxide.

**Figure 5:**
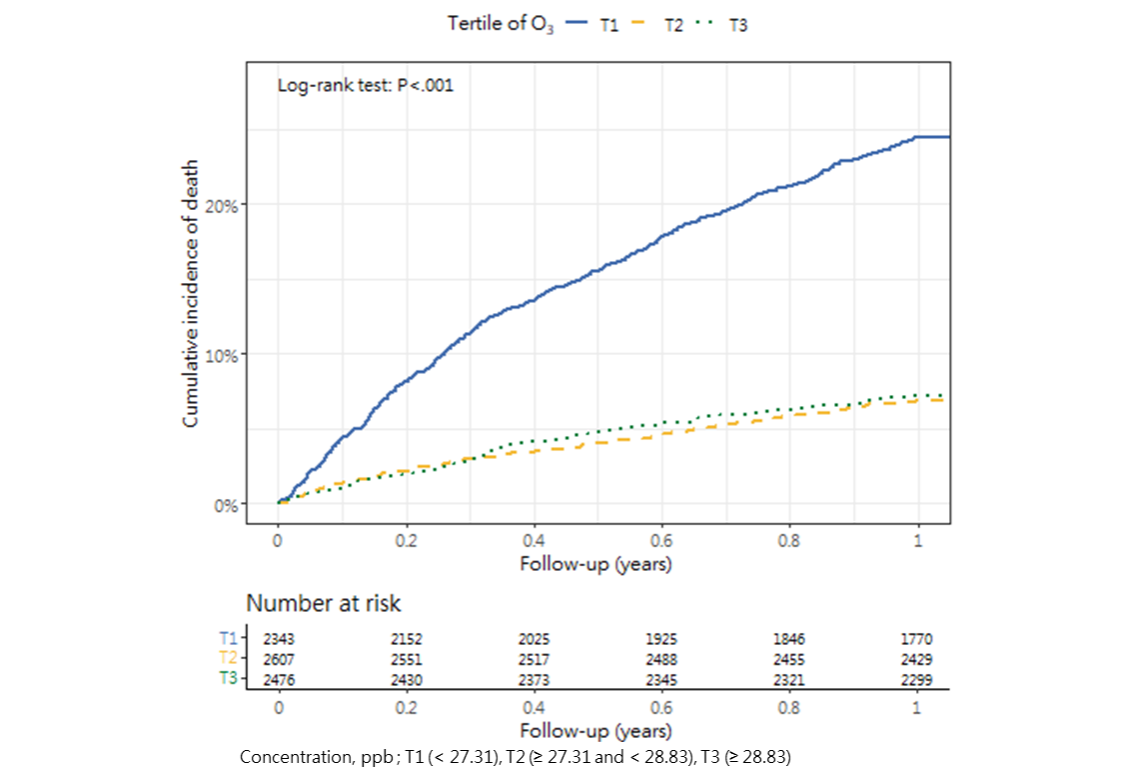
Cumulative incidence curves of mortality across tertiles of ozone.

**Figure 6:**
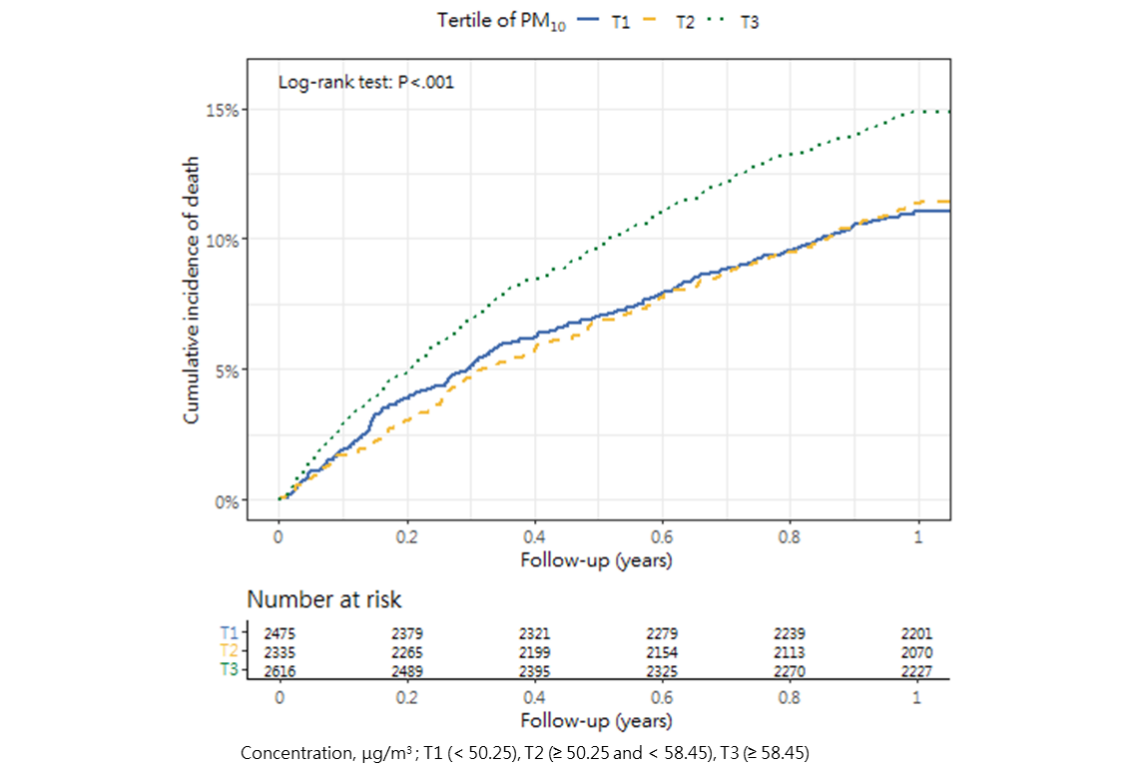
Cumulative incidence curves of mortality across tertiles of particulate matters having a size of <10 μm.

**Figure 7:**
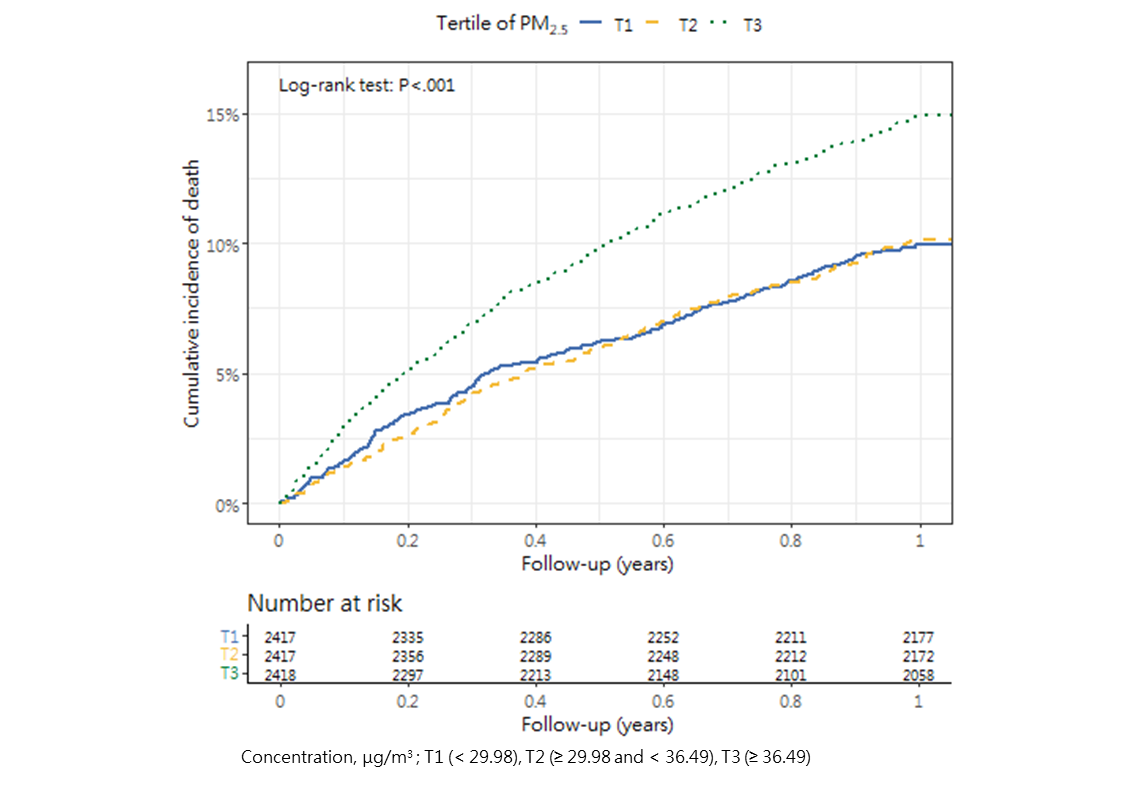
Cumulative incidence curves of mortality across tertiles of particulate matters having a size of <2.5 μm.

**Figure 8:**
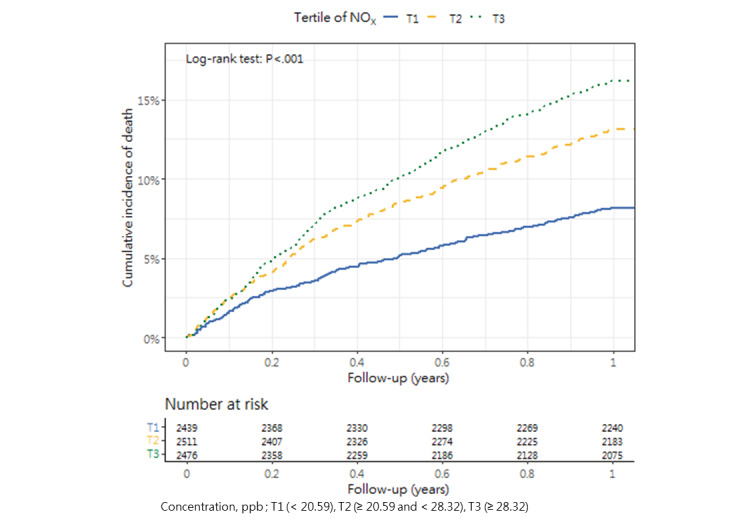
Cumulative incidence curves of mortality across tertiles of nitrogen oxides.

**Figure 9:**
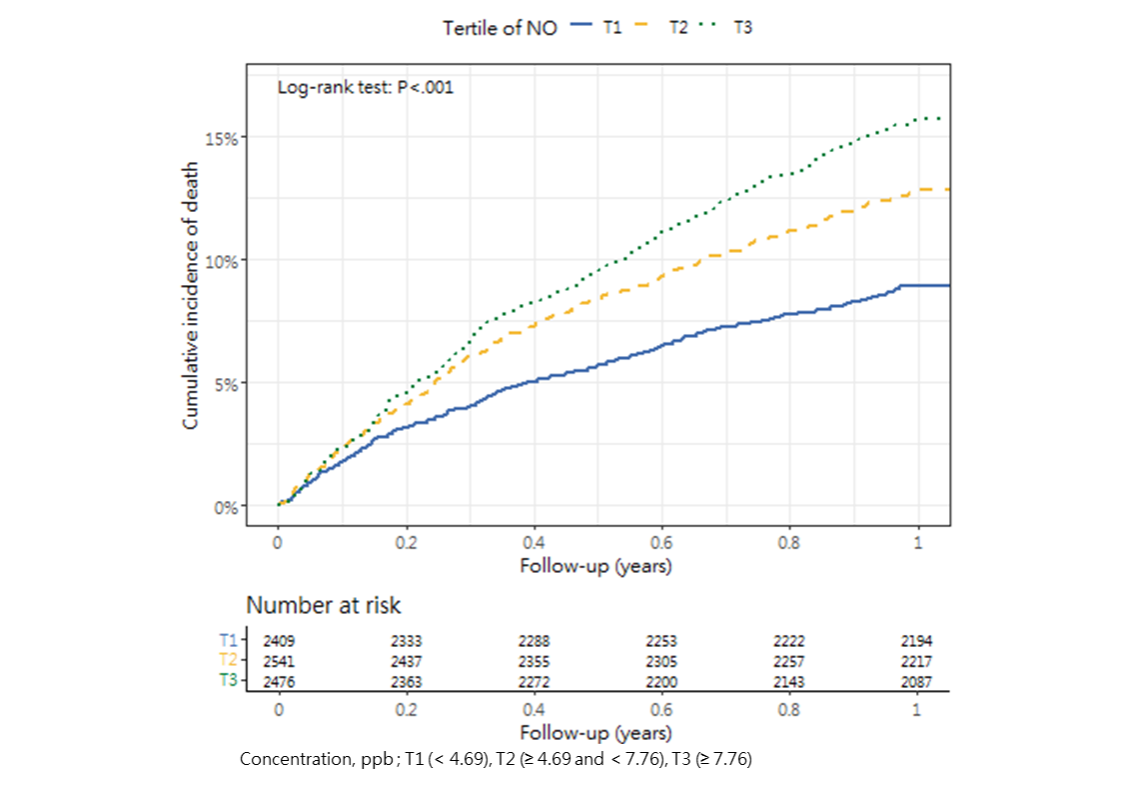
Cumulative incidence curves of mortality across tertiles of nitrogen monoxide.

**Figure 10:**
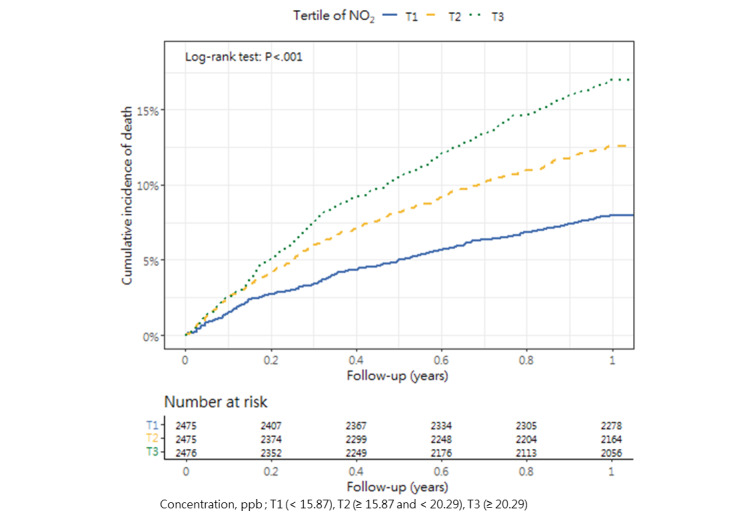
Cumulative incidence curves of mortality across tertiles of nitrogen dioxide.

**Figure 11:**
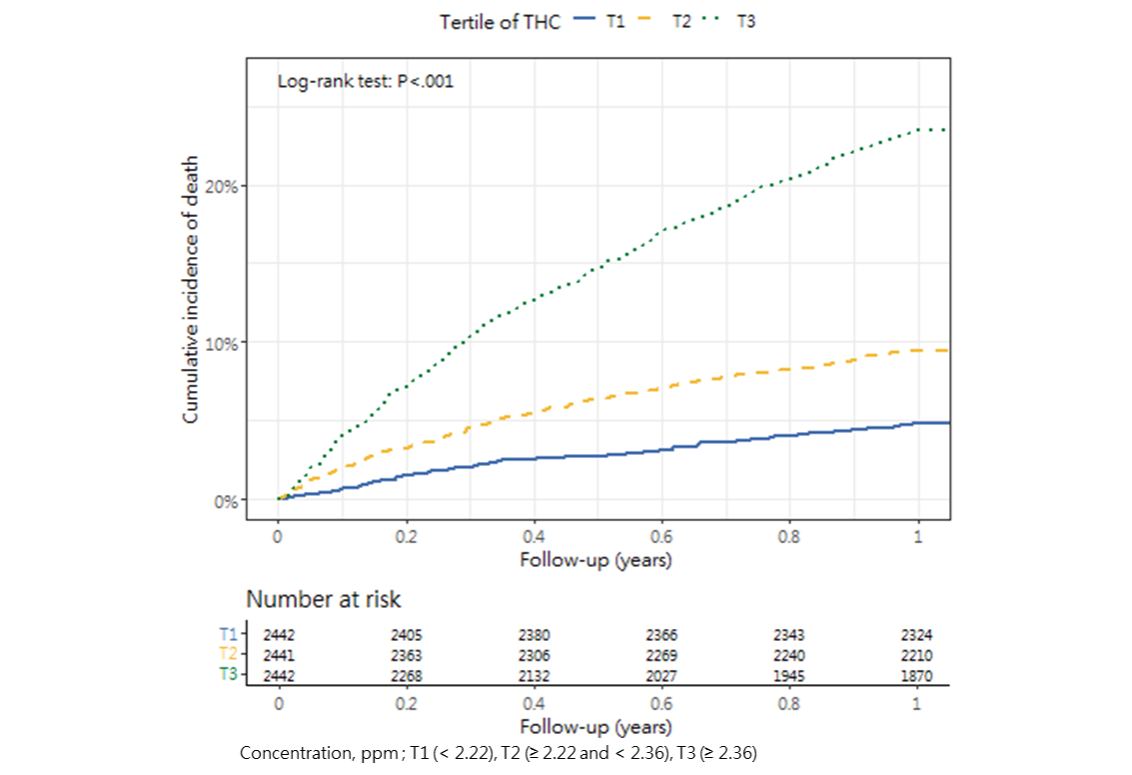
Cumulative incidence curves of mortality across tertiles of total hydrocarbons.

**Figure 12:**
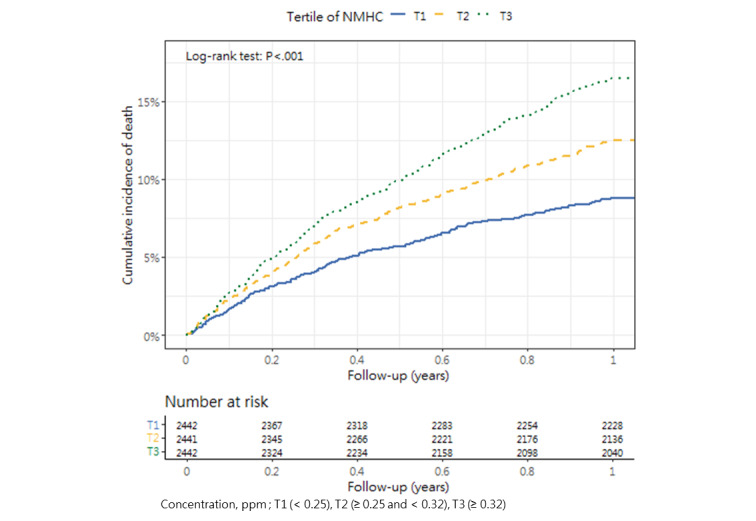
Cumulative incidence curves of mortality across tertiles of nonmethane hydrocarbons.

**Figure 13:**
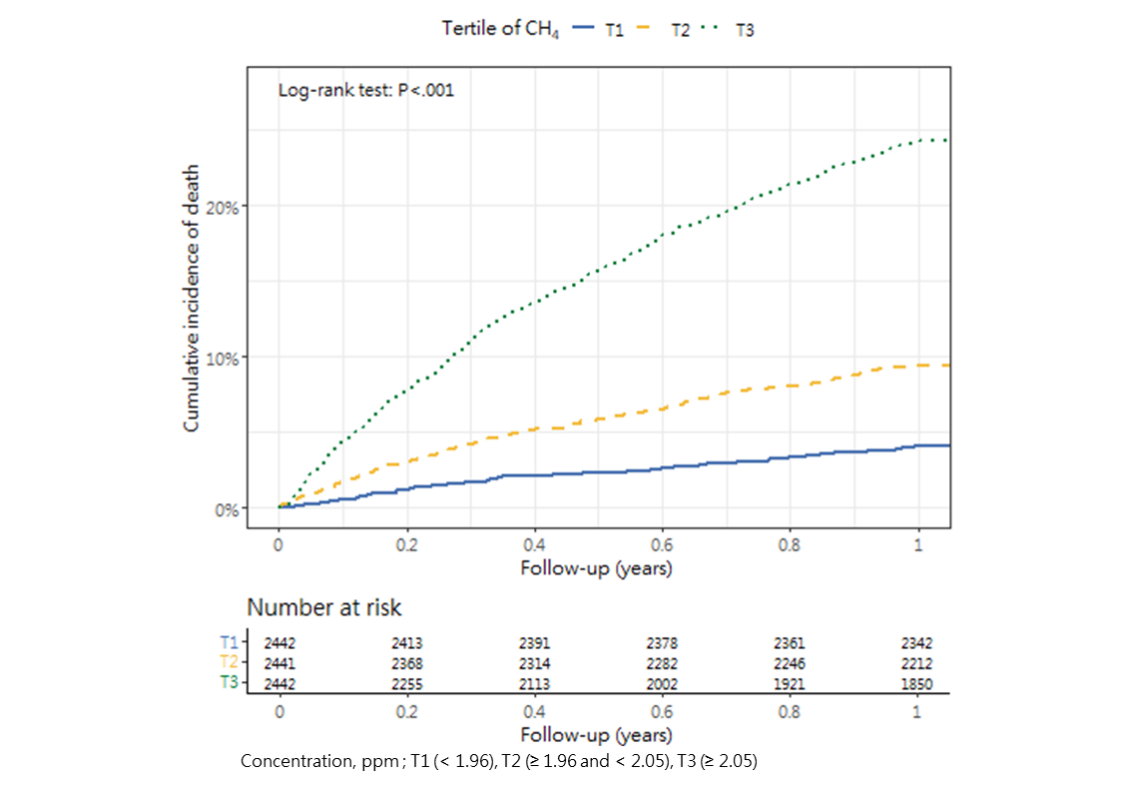
Cumulative incidence curves of mortality across tertiles of methane.

### Dose-Response Effects Between Air Pollutants and Mortality Risk

To evaluate the dose-response effects between the levels of air pollutants and the risk of mortality, we used Cox regression models and calculated the HRs for exposure at 1 SD increment for 10 years. Table 4 presents the corresponding HRs. The findings suggest that a 1 SD increment in the average exposure levels of SO_2_, CO, PM_10_, PM_2.5_, NO_X_, NO, NO_2_, THC, NMHC, and CH_4_ was associated with 14%, 49%, 18%, 12%, 41%, 33%, 38%, 20%, 9%, and 26% significant increases in mortality risk, respectively. However, a significantly negative association was observed for O_3_, with a 35% reduction in mortality risk. CO_2_ tended to exhibit only a trend but not a statistical significance to reduced mortality risk.

**Table 4:** Hazard ratios for mortality associated with long-term exposure to air pollution at 1 SD increment for 10 years.

Pollutants	Adjusted^a^ HR^b^ (95% CI)	*P* values	Mean (SD)
SO_2_^c^ (ppb^d^)	1.14 (1.06-1.23)	<.001	4.14 (1.35)
CO_2_^e^ (ppm^f^)	0.88 (0.76-1.02)	.09	398.46 (11.50)
CO^g^ (ppm)	1.49 (1.38-1.59)	<.001	0.55 (0.14)
O_3_^h^ (ppb)	0.65 (0.60-0.69)	<.001	28.08 (2.56)
PM_10_^i^ (μg/m^3^)	1.18 (1.08-1.29)	<.001	55.68 (9.78)
PM_2.5_^j^ (μg/m^3^)	1.12 (1.01-1.23)	.03	33.7 (6.85)
NO_X_^k^ (ppb)	1.41 (1.29-1.53)	<.001	25.43 (8.16)
NO^l^ (ppb)	1.33 (1.23-1.44)	<.001	7.41 (4.41)
NO_2_^m^ (ppb)	1.38 (1.26-1.50)	<.001	18.02 (4.16)
THC^n^ (ppm)	1.20 (1.11-1.29)	<.001	2.31 (0.17)
NMHC^o^ (ppm)	1.09 (1.01-1.17)	.03	0.30 (0.11)
CH_4_^p^ (ppm)	1.26 (1.16-1.36)	<.001	2.01 (0.11)

^a^Cox regression models were adjusted for age, urbanization level, insurance amount, Charlson Comorbidity Index score, hip fracture procedure, comedications, antiosteoporosis medication, ambient temperature, season, and lag 0-1.

^b^HR: hazard ratio.

^c^SO_2_: sulfur dioxide.

^d^ppb: parts per billion.

^e^CO_2_: carbon dioxide.

^f^ppm: parts per million.

^g^CO: carbon monoxide.

^h^O_3_: ozone.

^i^PM_10_: particulate matters having a size of <10 μm.

^j^PM_2.5_: particulate matters having a size of <2.5 μm.

^k^NO_X_: nitrogen oxides.

^l^NO: nitrogen monoxide.

^m^NO_2_: nitrogen dioxide.

^n^THC: total hydrocarbons.

^o^NMHC: nonmethane hydrocarbons.

^p^CH_4_: methane.

## Discussion

### Principal Findings

In this study, long-term exposure to SO_2_, CO, PM_10_, PM_2.5_, NO_X_, NO, NO_2_, THC, NMHC, and CH_4_ was associated with an increased risk of all-cause mortality within 1 year after hip fracture surgery. In older individuals, a 1 SD increment in the average exposure levels of SO_2_, CO, PM_10_, PM_2.5_, NO_X_, NO, NO_2_, THC, NMHC, and CH_4_ was associated with 14%, 49%, 18%, 12%, 41%, 33%, 38%, 20%, 9%, and 26% increases in mortality risk, respectively. By contrast, we found a significant reduction (35%) in the HR for O_3_ and a trend of potentially protective effects of CO_2_.

### Comparison With Prior Work

In this study, long-term exposure to SO_2_, CO, PM_10_, PM_2.5_, NO_X_, NO, NO_2_, THC, NMHC, and CH_4_ was consistently found to be risk factors for mortality within 1 year after hip fracture surgery. Previous studies have found an association between osteoporosis and the levels of exposure to air pollutants, including PM_2.5_, PM_10_, CO, and NO_2_ [62-65]. The same trends were observed in the risk of hip fracture associated with PM_2.5_, SO_2_, NO, NO_2_, and O_3_ [66,67]. Shi et al [35] were the first to investigate the effects of air pollution on mortality after hip fracture. In their nationwide cohort study in the United Kingdom, the levels of specific air pollutants, such as PM_2.5_, PM_10_, and NO_2_ exhibited a moderately positive association with the increases in the incidence and 30-day mortality rates of hip fracture. Thus far, their study was the only one on the negative effects of air pollution on mortality after hip fracture. However, the focus on 30-day mortality limited the scope of the long-term prognosis assessment. Moreover, the representativeness and extrapolation of their findings were limited because of the lack of age-standardization of the incidence; the possibility of errors in air pollution assessment owing to the use of data acquired only from the hospital region; and the presence of key unadjusted confounders, such as sex, season, temperature, socioeconomic, and CCI score. The aforementioned limitations of their study and the complexity of air pollution resulted in an unclear dose-response effect. To overcome this problem, we analyzed many pollutants; the robust evidence obtained in this study may help policy makers assess the economic loss and burden associated with air pollution and devise effective prevention strategies.

### Possible Mechanisms

Although the precise mechanisms underlying the association between air pollution and mortality remain unclear, we hypothesized the following mechanism: air pollution contributes to frailty in older adults, thus increasing their susceptibility to mortality after hip fracture surgery. Air pollution is an independent risk factor for mortality from respiratory and cardiac diseases [25,68]. Furthermore, air pollution modifies the aging process by interfering with biological pathways; these effects are supported by toxicological evidence indicating that air pollution enhances oxidative stress [43-52], activates systemic inflammation [36-42], and causes metabolic disorders [69-72], genetic and epigenetic alterations [73-81], and vector-mediated pathogen transmission [30]. Zanobetti and Schwartz [82] suggested that air pollution influences the frail population in the following manners: increasing the mortality rate, increasing recruitment into the frail group, and delaying recovery. This finding indicates that air pollution modifies aging through frailty. Moreover, air pollution may aggravate age-related decline and functional deterioration at the cellular, tissue, and organ levels [83], thus increasing individuals’ vulnerability to disease development and mortality incidence. Given the association of exposure to ambient air pollution with all-cause and specific morbidity and mortality, our hypothesis and the obtained significant HRs appear to be highly conceivable and concomitant.

We found a negative association between the risk of mortality after hip fracture and exposure to O_3_ pollution. Various conclusions have been reported regarding the effects of O_3_ exposure [84-88]. Studies reporting protective effects, which are consistent with our findings, have provided the following explanations. First, sunlight facilitates O_3_ formation; O_3_ is a seasonal pollutant because its level is higher in summer than in winter. Therefore, seasonal effects should be considered when analyzing the effects of O_3_ on health [84,89]. In this study, season served as a confounder in our regression models for potentially adjusting for the seasonal effects. Second, O_3_ is highly reactive and leads to the formation of other pollutants, such as NO_2_ and particulate matter [84,87]. Thus, O_3_ appears to be associated with other air pollutants; hence, the effects of O_3_ on health should be considered together with those of other pollutants. Thus, in this study, we performed a Pearson correlation analysis of air pollutants (Multimedia Appendix 1). To address the confounding impact of other pollutants and to prevent potential issues related to multicollinearity, we used potential associations among air pollutants to evaluate the impacts of multiple pollutants, selectively regulating those based on their weak correlations with other air pollutants. We defined the absolute value of correlation coefficients of <0.3 to indicate a low strength of correlation. However, the negative association between mortality and O_3_ pollution persisted (Multimedia Appendix 1) in the multiple-pollutant models of the targeted pollutants; thus, the second explanation was rejected. Thus, the precise mechanisms underlying the protective effects of O_3_ remain unclear. Nevertheless, other theories may provide some insights into these mechanisms, as follows: the enhanced expression levels of endothelial and inducible nitric oxide synthase [90-92], improvement of hemorheological parameters and oxygen delivery [92,93], and neuroprotective effects [92,94].

CO_2_ emission exhibits a nonsignificant negative correlation with mortality after hip fracture. For the correlation between exposure to CO_2_ pollution and the risk of mortality within 1 year after hip fracture surgery, the HR was 0.88 (95% CI 0.76-1.02), with a *P* value of .09. This finding is similar to previous findings that CO_2_ emission exerted positive but nonsignificant effects on longevity [95-97]. After adjustment for the urbanization level and insurance amount, our analysis revealed a positive but nonsignificant trend in the health effects of CO_2_ exposure, which is consistent with the literature. However, to the best of our knowledge, this has so far not been explained nor recognized in the literature. The need for more extensive research in the future is evident, as there remain numerous unexplored facets and complexities within this subject that warrant thorough investigation to advance our knowledge and drive progress in the field.

### Strengths

Our study has several strengths. First, to the best of our knowledge, this study is the first to indicate that long-term exposure to air pollution contributes to mortality within 1 year after hip fracture surgery. Second, we analyzed many key air pollutants (n=12). Third, we assessed the mortality within 1 year of surgery, thus focusing on the long-term outcomes of hip fracture. Finally, we adjusted for most of the major confounders, such as sex, season, temperature, socioeconomic, and CCI score, which further strengthened our findings and provided robustness for our primary conclusion.

### Limitations

This study has some limitations. First, although smoking is a key factor influencing ambient air quality, we could not include it in the analysis because of data unavailability; this might have led to a potential bias. Second, the NHIRD does not include data on disease severity, clinical manifestations, laboratory findings, and mortality causes. Hence, we could not determine the specific causes of mortality. Third, the possibility of errors in the assessment of air pollution cannot be ignored. Fourth, because we regarded death as withdrawal from the National Health Insurance program, bias might have been introduced into our findings. Fifth, while we considered any changes in residence during the assessment period, it is worth noting that the residential postcodes may not always be updated accurately in the insurance registration data. This could result in inaccuracies when reflecting patient characteristics. Sixth, although we adjusted for several confounders, unmeasured or unknown confounders might have introduced bias into our results. Given the limitations of retrospective health insurance database cohorts, which may not encompass all potential confounding factors, the possibility of residual bias due to unmeasured variables remains. Seventh, although we aimed to analyze individual molecules separately, it is possible that we neglected the potential for nonlinear relationships or the possibility of mixed effects of other molecules. Finally, there is a potential that the current levels of air pollution may differ from the data used in our study. However, due to the availability of data, this is the most up-to-date information we can analyze. Furthermore, our paper highlights the health and societal hazards of air pollution, and we hope that our research findings can provide empirical support for air pollution control policies across various times and situations. Despite the aforementioned limitations, this study provides adequate, high-quality evidence to support our conclusion that long-term exposure to SO_2_, CO, PM_10_, PM_2.5_, NO_X_, NO, NO_2_, THC, NMHC, and CH_4_ is associated with an increased risk of all-cause mortality within 1 year after hip fracture surgery.

### Conclusions

In this nationwide population-based retrospective study, we discovered that long-term exposure to SO_2_, CO, PM_10_, PM_2.5_, NO_X_, NO, NO_2_, THC, NMHC, and CH_4_ is correlated with an increased risk of all-cause mortality within 1 year after hip fracture surgery in Taiwanese adults older than 60 years. Furthermore, 1 SD increment in the average exposure levels of SO_2_, CO, PM_10_, PM_2.5_, NO_X_, NO, NO_2_, THC, NMHC, and CH_4_ was associated with 14%, 49%, 18%, 12%, 41%, 33%, 38%, 20%, 9%, and 26% increases in mortality risk, respectively. However, O_3_ exposure was associated with a significant reduction (35%) in HR, whereas CO_2_ exhibited a nonsignificant trend of potentially protective effects. Thus, our findings provide robust evidence that can be used by the government to devise and implement air pollution prevention strategies for reducing the burden on the health care system. Nonetheless, future studies with well-adjusted confounders are warranted to investigate the correlation between air pollution and hip fracture mortality and the underlying pathophysiologic mechanisms.

## Appendix

Multimedia Appendix 1 The International Classification of Diseases, Ninth Revision, (ICD-9) codes and Anatomical Therapeutic Chemical (ATC) Classification code established in the inclusion criteria, exclusion criteria, and definition of confounders.

Multimedia Appendix 2 Characteristics of the study population across the tertiles of SO_2_ exposure.

Multimedia Appendix 3 Characteristics of the study population across the tertiles of CO_2_ exposure.

Multimedia Appendix 4 Characteristics of the study population across the tertiles of CO exposure.

Multimedia Appendix 5 Characteristics of the study population across the tertiles of O_3_ exposure.

Multimedia Appendix 6 Characteristics of the study population across the tertiles of PM_10_ exposure.

Multimedia Appendix 7 Characteristics of the study population across the tertiles of PM_2.5_ exposure.

Multimedia Appendix 8 Characteristics of the study population across the tertiles of NO_X_ exposure.

Multimedia Appendix 9 Characteristics of the study population across the tertiles of NO exposure.

Multimedia Appendix 10 Characteristics of the study population across the tertiles of NO_2_ exposure.

Multimedia Appendix 11 Characteristics of the study population across the tertiles of THC exposure.

Multimedia Appendix 12 Characteristics of the study population across the tertiles of NMHC exposure.

Multimedia Appendix 13 Characteristics of the study population across the tertiles of CH_4_ exposure.

Multimedia Appendix 14 Pearson correlation analysis for air pollutants detected over the exposure period.

Multimedia Appendix 15 Hazard ratios for long-term ozone exposure at 1 standard deviation increment associated with mortality rate.

## Abbreviations

CCI: Charlson Comorbidity Index
CH_4_: methane
CO: carbon monoxide
CO_2_: carbon dioxide
EPA: Environmental Protection Administration
HR: hazard ratio
ICD-9: International Classification of Diseases, Ninth Revision
NHIRD: National Health Insurance Research Database
NMHC: nonmethane hydrocarbons
NO: nitrogen monoxide
NO_2_: nitrogen dioxide
NO_X_: nitrogen oxides
O_3_: ozone
PM_10_: particulate matters having a size of <10 μm
PM_2.5_: particulate matters having a size of <2.5 μm
SO_2_: sulfur dioxide
THC: total hydrocarbons

## Data Availability

All data generated or analyzed during this study are included in this published paper and its Multimedia Appendix files. More detailed data sets are not publicly accessible due to the requirement of obtaining approval from the Taiwan Ministry of Health and Welfare. Researchers interested in obtaining access to this data set may initiate the process by submitting an application form to the Ministry of Health and Welfare. For further guidance and assistance, please reach out to the MOHW staff via email at stcarolwu@mohw.gov.tw. The address of the Taiwan Ministry of Health and Welfare is as follows: No.488, Sec. 6, Zhongxiao E. Rd., Nangang Dist., Taipei City 115, Taiwan. You can also contact them by phone at +886-2-8590-6848.

## References

[1] Guzon-Illescas O, Fernandez EP, Villarias NC, Donate FJQ, Peña M, Alonso-Blas C, García-Vadillo A, Mazzucchelli R (2019). Mortality after osteoporotic hip fracture: incidence, trends, and associated factors. J Orthop Surg Res.

[2] LeBlanc KE, Muncie HL, LeBlanc LL (2014). Hip fracture: diagnosis, treatment, and secondary prevention. Am Fam Physician.

[3] Aarden JJ, van der Esch M, Engelbert RHH, van der Schaaf M, de Rooij SE, Buurman BM (2017). Hip fractures in older patients: trajectories of disability after surgery. J Nutr Health Aging.

[4] Chen YP, Kuo YJ, Liu CH, Chien PC, Chang WC, Lin CY, Pakpour AH (2021). Prognostic factors for 1-year functional outcome, quality of life, care demands, and mortality after surgery in Taiwanese geriatric patients with a hip fracture: a prospective cohort study. Ther Adv Musculoskelet Dis.

[5] Kammerlander C, Gosch M, Kammerlander-Knauer U, Luger TJ, Blauth M, Roth T (2011). Long-term functional outcome in geriatric hip fracture patients. Arch Orthop Trauma Surg.

[6] Makridis KG, Karachalios T, Kontogeorgakos VA, Badras LS, Malizos KN (2015). The effect of osteoporotic treatment on the functional outcome, re-fracture rate, quality of life and mortality in patients with hip fractures: a prospective functional and clinical outcome study on 520 patients. Injury.

[7] Peeters CMM, Visser E, Van de Ree CLP, Gosens T, Den Oudsten BL, De Vries J (2016). Quality of life after hip fracture in the elderly: a systematic literature review. Injury.

[8] Rosell PAE, Parker MJ (2003). Functional outcome after hip fracture. a 1-year prospective outcome study of 275 patients. Injury.

[9] Chiang MH, Huang YY, Kuo YJ, Huang SW, Jang YC, Chu FL, Chen YP (2022). Prognostic factors for mortality, activity of daily living, and quality of life in Taiwanese older patients within 1 year following hip fracture surgery. J Pers Med.

[10] Browner WS, Pressman AR, Nevitt MC, Cummings SR (1996). Mortality following fractures in older women. the study of osteoporotic fractures. Arch Intern Med.

[11] Center JR, Nguyen TV, Schneider D, Sambrook PN, Eisman JA (1999). Mortality after all major types of osteoporotic fracture in men and women: an observational study. Lancet.

[12] Cummings SR, Melton LJ (2002). Epidemiology and outcomes of osteoporotic fractures. Lancet.

[13] Kanis JA, Oden A, Johnell O, De Laet C, Jonsson B, Oglesby AK (2003). The components of excess mortality after hip fracture. Bone.

[14] Tarazona-Santabalbina FJ, Belenguer-Varea A, Rovira-Daudi E, Salcedo-Mahiques E, Cuesta-Peredó D, Doménech-Pascual JR, Salvador-Pérez MI, Avellana-Zaragoza JA (2012). Early interdisciplinary hospital intervention for elderly patients with hip fractures : functional outcome and mortality. Clinics (Sao Paulo).

[15] Chen YP, Chang WC, Wen TW, Chien PC, Huang SW, Kuo YJ (2022). Multipronged programmatic strategy for preventing secondary fracture and facilitating functional recovery in older patients after hip fractures: our experience in Taipei municipal wanfang hospital. Medicina (Kaunas).

[16] Barceló M, Torres OH, Mascaró J, Casademont J (2021). Hip fracture and mortality: study of specific causes of death and risk factors. Arch Osteoporos.

[17] Panula J, Pihlajamäki H, Mattila VM, Jaatinen P, Vahlberg T, Aarnio P, Kivelä SL (2011). Mortality and cause of death in hip fracture patients aged 65 or older: a population-based study. BMC Musculoskelet Disord.

[18] von Friesendorff M, McGuigan FE, Wizert A, Rogmark C, Holmberg AH, Woolf AD, Akesson K (2016). Hip fracture, mortality risk, and cause of death over two decades. Osteoporos Int.

[19] Wehren LE, Hawkes WG, Orwig DL, Hebel JR, Zimmerman SI, Magaziner J (2003). Gender differences in mortality after hip fracture: the role of infection. J Bone Miner Res.

[20] Morri M, Ambrosi E, Chiari P, Magli AO, Gazineo D, D'Alessandro F, Forni C (2019). One-year mortality after hip fracture surgery and prognostic factors: a prospective cohort study. Sci Rep.

[21] Roche JJW, Wenn RT, Sahota O, Moran CG (2005). Effect of comorbidities and postoperative complications on mortality after hip fracture in elderly people: prospective observational cohort study. BMJ.

[22] Xu BY, Yan S, Low LL, Vasanwala FF, Low SG (2019). Predictors of poor functional outcomes and mortality in patients with hip fracture: a systematic review. BMC Musculoskelet Disord.

[23] Cohen AJ, Brauer M, Burnett R, Anderson HR, Frostad J, Estep K, Balakrishnan K, Brunekreef B, Dandona L, Dandona R, Feigin V, Freedman G, Hubbell B, Jobling A, Kan H, Knibbs L, Liu Y, Martin R, Morawska L, Pope CA, Shin H, Straif K, Shaddick G, Thomas M, van Dingenen R, van Donkelaar A, Vos T, Murray CJL, Forouzanfar MH (2017). Estimates and 25-year trends of the global burden of disease attributable to ambient air pollution: an analysis of data from the global burden of diseases study 2015. Lancet.

[24] Dockery DW, Pope CA, Xu X, Spengler JD, Ware JH, Fay ME, Ferris BG, Speizer FE (1993). An association between air pollution and mortality in six U.S. cities. N Engl J Med.

[25] Liu C, Chen R, Sera F, Vicedo-Cabrera AM, Guo Y, Tong S, Coelho MSZS, Saldiva PHN, Lavigne E, Matus P, Ortega NV, Garcia SO, Pascal M, Stafoggia M, Scortichini M, Hashizume M, Honda Y, Hurtado-Díaz M, Cruz J, Nunes B, Teixeira JP, Kim H, Tobias A, Íñiguez C, Forsberg B, Åström C, Ragettli MS, Guo YL, Chen BY, Bell ML, Wright CY, Scovronick N, Garland RM, Milojevic A, Kyselý J, Urban A, Orru H, Indermitte E, Jaakkola JJK, Ryti NRI, Katsouyanni K, Analitis A, Zanobetti A, Schwartz J, Chen J, Wu T, Cohen A, Gasparrini A, Kan H (2019). Ambient particulate air pollution and daily mortality in 652 cities. N Engl J Med.

[26] Samet JM, Dominici F, Curriero FC, Coursac I, Zeger SL (2000). Fine particulate air pollution and mortality in 20 U.S. cities, 1987-1994. N Engl J Med.

[27] Coleman NC, Burnett RT, Higbee JD, Lefler JS, Merrill RM, Ezzati M, Marshall JD, Kim SY, Bechle M, Robinson AL, Pope CA (2020). Cancer mortality risk, fine particulate air pollution, and smoking in a large, representative cohort of US adults. Cancer Causes Control.

[28] Im C, Kim DH, Oh TK (2020). Preadmission exposure to air pollution and 90-day mortality in critically ill patients: a retrospective study. J Occup Environ Med.

[29] Shetty BSP, D'Souza G, Anand MP (2021). Effect of indoor air pollution on Chronic Obstructive Pulmonary Disease (COPD) deaths in Southern Asia-a systematic review and meta-analysis. Toxics.

[30] Comunian S, Dongo D, Milani C, Palestini P (2020). Air pollution and COVID-19: the role of particulate matter in the spread and increase of COVID-19's morbidity and mortality. Int J Environ Res Public Health.

[31] Al-Kindi SG, Sarode A, Zullo M, Brook J, Burnett R, Oliveira GH, Huang W, Brook R, Rajagopalan S (2019). Ambient air pollution and mortality after cardiac transplantation. J Am Coll Cardiol.

[32] Feng Y, Jones MR, Ahn JB, Garonzik-Wang JM, Segev DL, McAdams-DeMarco M (2021). Ambient air pollution and posttransplant outcomes among kidney transplant recipients. Am J Transplant.

[33] Ruttens D, Verleden SE, Bijnens EM, Winckelmans E, Gottlieb J, Warnecke G, Meloni F, Morosini M, Van Der Bij W, Verschuuren EA, Sommerwerck U, Weinreich G, Kamler M, Roman A, Gomez-Olles S, Berastegui C, Benden C, Holm AM, Iversen M, Schultz HH, Luijk B, Oudijk EJ, Kwakkel-van Erp JM, Jaksch P, Klepetko W, Kneidinger N, Neurohr C, Corris P, Fisher AJ, Lordan J, Meachery G, Piloni D, Vandermeulen E, Bellon H, Hoffmann B, Vienneau D, Hoek G, de Hoogh K, Nemery B, Verleden GM, Vos R, Nawrot TS, Vanaudenaerde BM (2017). An association of particulate air pollution and traffic exposure with mortality after lung transplantation in Europe. Eur Respir J.

[34] Liu JJ, Fu SB, Jiang J, Tang XL (2021). Association between outdoor particulate air pollution and the risk of osteoporosis: a systematic review and meta-analysis. Osteoporos Int.

[35] Shi W, Huang C, Chen S, Yang C, Liu N, Zhu X, Su X, Zhu X, Lin J (2022). Long-term exposure to air pollution increases hip fracture incidence rate and related mortality: analysis of national hip fracture database. Osteoporos Int.

[36] Brook RD, Rajagopalan S, Pope CA, Brook JR, Bhatnagar A, Diez-Roux AV, Holguin F, Hong Y, Luepker RV, Mittleman MA, Peters A, Siscovick D, Smith SC, Whitsel L, Kaufman JD (2010). Particulate matter air pollution and cardiovascular disease: an update to the scientific statement from the American Heart Association. Circulation.

[37] Sun Q, Wang A, Jin X, Natanzon A, Duquaine D, Brook RD, Aguinaldo JGS, Fayad ZA, Fuster V, Lippmann M, Chen LC, Rajagopalan S (2005). Long-term air pollution exposure and acceleration of atherosclerosis and vascular inflammation in an animal model. JAMA.

[38] Delfino RJ, Staimer N, Tjoa T, Polidori A, Arhami M, Gillen DL, Kleinman MT, Vaziri ND, Longhurst J, Zaldivar F, Sioutas C (2008). Circulating biomarkers of inflammation, antioxidant activity, and platelet activation are associated with primary combustion aerosols in subjects with coronary artery disease. Environ Health Perspect.

[39] Hoffmann B, Moebus S, Dragano N, Stang A, Möhlenkamp S, Schmermund A, Memmesheimer M, Bröcker-Preuss M, Mann K, Erbel R, Jöckel KH (2009). Chronic residential exposure to particulate matter air pollution and systemic inflammatory markers. Environ Health Perspect.

[40] Panasevich S, Leander K, Rosenlund M, Ljungman P, Bellander T, de Faire U, Pershagen G, Nyberg F (2009). Associations of long- and short-term air pollution exposure with markers of inflammation and coagulation in a population sample. Occup Environ Med.

[41] Rückerl R, Greven S, Ljungman P, Aalto P, Antoniades C, Bellander T, Berglind N, Chrysohoou C, Forastiere F, Jacquemin B, von Klot S, Koenig W, Küchenhoff H, Lanki T, Pekkanen J, Perucci CA, Schneider A, Sunyer J, Peters A (2007). Air pollution and inflammation (interleukin-6, C-reactive protein, fibrinogen) in myocardial infarction survivors. Environ Health Perspect.

[42] Rückerl R, Schneider A, Breitner S, Cyrys J, Peters A (2011). Health effects of particulate air pollution: a review of epidemiological evidence. Inhal Toxicol.

[43] Tsai DH, Riediker M, Berchet A, Paccaud F, Waeber G, Vollenweider P, Bochud M (2019). Effects of short- and long-term exposures to particulate matter on inflammatory marker levels in the general population. Environ Sci Pollut Res Int.

[44] Andersson H, Piras E, Demma J, Hellman B, Brittebo E (2009). Low levels of the air pollutant 1-nitropyrene induce DNA damage, increased levels of reactive oxygen species and endoplasmic reticulum stress in human endothelial cells. Toxicology.

[45] Bai Y, Suzuki AK, Sagai M (2001). The cytotoxic effects of diesel exhaust particles on human pulmonary artery endothelial cells in vitro: role of active oxygen species. Free Radic Biol Med.

[46] Ghio AJ, Kim C, Devlin RB (2000). Concentrated ambient air particles induce mild pulmonary inflammation in healthy human volunteers. Am J Respir Crit Care Med.

[47] Ghio AJ, Richards JH, Carter JD, Madden MC (2000). Accumulation of iron in the rat lung after tracheal instillation of diesel particles. Toxicol Pathol.

[48] Hirano S, Furuyama A, Koike E, Kobayashi T (2003). Oxidative-stress potency of organic extracts of diesel exhaust and urban fine particles in rat heart microvessel endothelial cells. Toxicology.

[49] Jaeschke H (2011). Reactive oxygen and mechanisms of inflammatory liver injury: present concepts. J Gastroenterol Hepatol.

[50] Park JH, Troxel AB, Harvey RG, Penning TM (2006). Polycyclic Aromatic Hydrocarbon (PAH) o-quinones produced by the Aldo-Keto-Reductases (AKRs) generate abasic sites, oxidized pyrimidines, and 8-oxo-dGuo via reactive oxygen species. Chem Res Toxicol.

[51] Risom L, Møller P, Loft S (2005). Oxidative stress-induced DNA damage by particulate air pollution. Mutat Res.

[52] Wold LE, Simkhovich BZ, Kleinman MT, Nordlie MA, Dow JS, Sioutas C, Kloner RA (2006). In vivo and in vitro models to test the hypothesis of particle-induced effects on cardiac function and arrhythmias. Cardiovasc Toxicol.

[53] Norring-Agerskov D, Bathum L, Pedersen OB, Abrahamsen B, Lauritzen JB, Jørgensen NR, Jørgensen HL (2019). Biochemical markers of inflammation are associated with increased mortality in hip fracture patients: the bispebjerg hip fracture biobank. Aging Clin Exp Res.

[54] Wang Z, Ehnert S, Ihle C, Schyschka L, Pscherer S, Nussler NC, Braun KF, Van Griensven M, Wang G, Burgkart R, Stöckle U, Gebhard F, Vester H, Nussler AK (2014). Increased oxidative stress response in granulocytes from older patients with a hip fracture may account for slow regeneration. Oxid Med Cell Longev.

[55] Simoni M, Baldacci S, Maio S, Cerrai S, Sarno G, Viegi G (2015). Adverse effects of outdoor pollution in the elderly. J Thorac Dis.

[56] Cheng CL, Kao YHY, Lin SJ, Lee CH, Lai ML (2011). Validation of the national health insurance research database with ischemic stroke cases in Taiwan. Pharmacoepidemiol Drug Saf.

[57] Hsing AW, Ioannidis JPA (2015). Nationwide population science: lessons from the Taiwan national health insurance research database. JAMA Intern Med.

[58] Liu CY, Hung YT, Chuang YL, Chen YJ, Weng WS, Liu JS, Liang KY (2006). 台灣地區鄉鎮市區發展類型應用於大型健康調查抽樣設計之研究 [Incorporating development stratification of Taiwan townships into sampling design of large scale health interview survey]. J Community Work Community Studies.

[59] Chen R, Kan H, Chen B, Huang W, Bai Z, Song G, Pan G (2012). Association of particulate air pollution with daily mortality: the China air pollution and health effects study. Am J Epidemiol.

[60] Chen R, Yin P, Meng X, Liu C, Wang L, Xu X, Ross JA, Tse LA, Zhao Z, Kan H, Zhou M (2017). Fine particulate air pollution and daily mortality. a nationwide analysis in 272 Chinese cities. Am J Respir Crit Care Med.

[61] Lee H, Honda Y, Hashizume M, Guo YL, Wu CF, Kan H, Jung K, Lim YH, Yi S, Kim H (2015). Short-term exposure to fine and coarse particles and mortality: a multicity time-series study in East Asia. Environ Pollut.

[62] Alvaer K, Meyer HE, Falch JA, Nafstad P, Søgaard AJ (2007). Outdoor air pollution and bone mineral density in elderly men—the oslo health study. Osteoporos Int.

[63] Alver K, Meyer HE, Falch JA, Søgaard AJ (2010). Outdoor air pollution, bone density and self-reported forearm fracture: the oslo health study. Osteoporos Int.

[64] Calderón-Garcidueñas L, Mora-Tiscareño A, Francolira M, Torres-Jardón R, Peña-Cruz B, Palacios-López C, Zhu H, Kong L, Mendoza-Mendoza N, Montesinoscorrea H, Romero L, Valencia-Salazar G, Kavanaugh M, Frenk S (2013). Exposure to urban air pollution and bone health in clinically healthy six-year-old children. Arh Hig Rada Toksikol.

[65] Chang KH, Chang MY, Muo CH, Wu TN, Hwang BF, Chen CY, Lin TH, Kao CH (2015). Exposure to air pollution increases the risk of osteoporosis: a nationwide longitudinal study. Medicine (Baltimore).

[66] Mazzucchelli R, Villarias NC, Fernandez EP, Reguera MLD, Garcia-Vadillo A, Quiros FJ, Guzon O, Caravaca GR, de Miguel AG (2018). Short-term association between outdoor air pollution and osteoporotic hip fracture. Osteoporos Int.

[67] Prada D, Zhong J, Colicino E, Zanobetti A, Schwartz J, Dagincourt N, Fang SC, Kloog I, Zmuda JM, Holick M, Herrera LA, Hou L, Dominici F, Bartali B, Baccarelli AA (2017). Association of air particulate pollution with bone loss over time and bone fracture risk: analysis of data from two independent studies. Lancet Planet Health.

[68] Requia WJ, Adams MD, Arain A, Papatheodorou S, Koutrakis P, Mahmoud M (2018). Global association of air pollution and cardiorespiratory diseases: a systematic review, meta-analysis, and investigation of modifier variables. Am J Public Health.

[69] Andersen ZJ, Raaschou-Nielsen O, Ketzel M, Jensen SS, Hvidberg M, Loft S, Tjønneland A, Overvad K, Sørensen M (2012). Diabetes incidence and long-term exposure to air pollution: a cohort study. Diabetes Care.

[70] Chuang KJ, Yan YH, Chiu SY, Cheng TJ (2011). Long-term air pollution exposure and risk factors for cardiovascular diseases among the elderly in Taiwan. Occup Environ Med.

[71] Egeland GM, Sweeney MH, Fingerhut MA, Wille KK, Schnorr TM, Halperin WE (1994). Total serum testosterone and gonadotropins in workers exposed to dioxin. Am J Epidemiol.

[72] Sun Z, Mukherjee B, Brook RD, Gatts GA, Yang F, Sun Q, Brook JR, Fan Z, Rajagopalan S (2013). Air-Pollution and Cardiometabolic Diseases (AIRCMD): a prospective study investigating the impact of air pollution exposure and propensity for type II diabetes. Sci Total Environ.

[73] Billet S, Abbas I, Le Goff J, Verdin A, André V, Lafargue PE, Hachimi A, Cazier F, Sichel F, Shirali P, Garçon G (2008). Genotoxic potential of polycyclic aromatic hydrocarbons-coated onto airborne Particulate Matter (PM 2.5) in human lung epithelial A549 cells. Cancer Lett.

[74] Bonetta S, Gianotti V, Bonetta S, Gosetti F, Oddone M, Gennaro MC, Carraro E (2009). DNA damage in A549 cells exposed to different extracts of PM(2.5) from industrial, urban and highway sites. Chemosphere.

[75] Dergham M, Lepers C, Verdin A, Billet S, Cazier F, Courcot D, Shirali P, Garçon G (2012). Prooxidant and proinflammatory potency of air pollution Particulate Matter (PM2.5-0.3) produced in rural, urban, or industrial surroundings in human bronchial epithelial cells (BEAS-2B). Chem Res Toxicol.

[76] Gilmour PS, Rahman I, Donaldson K, MacNee W (2003). Histone acetylation regulates epithelial IL-8 release mediated by oxidative stress from environmental particles. Am J Physiol Lung Cell Mol Physiol.

[77] Jiang L, Dai H, Sun Q, Geng C, Yang Y, Wu T, Zhang X, Zhong L (2011). Ambient particulate matter on DNA damage in HepG2 cells. Toxicol Ind Health.

[78] Ke Q, Davidson T, Chen H, Kluz T, Costa M (2006). Alterations of histone modifications and transgene silencing by nickel chloride. Carcinogenesis.

[79] Lepers C, André V, Dergham M, Billet S, Verdin A, Garçon G, Dewaele D, Cazier F, Sichel F, Shirali P (2014). Xenobiotic metabolism induction and bulky DNA adducts generated by particulate matter pollution in BEAS-2B cell line: geographical and seasonal influence. J Appl Toxicol.

[80] Lepers C, Dergham M, Armand L, Billet S, Verdin A, Andre V, Pottier D, Courcot D, Shirali P, Sichel F (2014). Mutagenicity and clastogenicity of native airborne particulate matter samples collected under industrial, urban or rural influence. Toxicol In Vitro.

[81] Perrone MG, Gualtieri M, Ferrero L, Lo Porto C, Udisti R, Bolzacchini E, Camatini M (2010). Seasonal variations in chemical composition and in vitro biological effects of fine PM from Milan. Chemosphere.

[82] Zanobetti A, Schwartz J (2008). Mortality displacement in the association of ozone with mortality: an analysis of 48 cities in the United States. Am J Respir Crit Care Med.

[83] Fedarko NS (2011). The biology of aging and frailty. Clin Geriatr Med.

[84] Al Ahad MA, Sullivan F, Demšar U, Melhem M, Kulu H (2020). The effect of air-pollution and weather exposure on mortality and hospital admission and implications for further research: a systematic scoping review. PLoS One.

[85] Atkinson RW, Butland BK, Dimitroulopoulou C, Heal MR, Stedman JR, Carslaw N, Jarvis D, Heaviside C, Vardoulakis S, Walton H, Anderson HR (2016). Long-term exposure to ambient ozone and mortality: a quantitative systematic review and meta-analysis of evidence from cohort studies. BMJ Open.

[86] Di Q, Wang Y, Zanobetti A, Wang Y, Koutrakis P, Choirat C, Dominici F, Schwartz JD (2017). Air pollution and mortality in the medicare population. N Engl J Med.

[87] Hvidtfeldt UA, Sørensen M, Geels C, Ketzel M, Khan J, Tjønneland A, Overvad K, Brandt J, Raaschou-Nielsen O (2019). Long-term residential exposure to PM, PM, black carbon, NO, and ozone and mortality in a Danish cohort. Environ Int.

[88] Turner MC, Jerrett M, Pope CA, Krewski D, Gapstur SM, Diver WR, Beckerman BS, Marshall JD, Su J, Crouse DL, Burnett RT (2016). Long-term ozone exposure and mortality in a large prospective study. Am J Respir Crit Care Med.

[89] Carey IM, Atkinson RW, Kent AJ, van Staa T, Cook DG, Anderson HR (2013). Mortality associations with long-term exposure to outdoor air pollution in a national English cohort. Am J Respir Crit Care Med.

[90] Ajamieh HH, Menéndez S, Martínez-Sánchez G, Candelario-Jalil E, Re L, Giuliani A, Fernández OSL (2004). Effects of ozone oxidative preconditioning on nitric oxide generation and cellular redox balance in a rat model of hepatic ischaemia-reperfusion. Liver Int.

[91] Chen H, Xing B, Liu X, Zhan B, Zhou J, Zhu H, Chen Z (2008). Ozone oxidative preconditioning protects the rat kidney from reperfusion injury: the role of nitric oxide. J Surg Res.

[92] Yu Y, Dong H, Yao S, Ji M, Yao X, Zhang Z (2017). Protective effects of ambient ozone on incidence and outcomes of ischemic stroke in Changzhou, China: a time-series study. Int J Environ Res Public Health.

[93] Giunta R, Coppola A, Luongo C, Sammartino A, Guastafierro S, Grassia A, Giunta L, Mascolo L, Tirelli A, Coppola L (2001). Ozonized autohemotransfusion improves hemorheological parameters and oxygen delivery to tissues in patients with peripheral occlusive arterial disease. Ann Hematol.

[94] Frosini M, Contartese A, Zanardi I, Travagli V, Bocci V (2012). Selective ozone concentrations may reduce the ischemic damage after a stroke. Free Radic Res.

[95] Amuka JI, Asogwa FO, Ugwuanyi RO, Omeje AN, Onyechi T (2018). Climate change and life expectancy in a developing countryvidence from greenhouse gas (CO2) emission in Nigeria. Int J Econ Financ Issues.

[96] Delavari S, Zandian H, Rezaei S, Moradinazar M, Delavari S, Saber A, Fallah R (2016). Life expectancy and its socioeconomic determinants in Iran. Electron Physician.

[97] Monsef A, Mehrjardi AS (2015). Determinants of life expectancy: a panel data approach. Asian Econ Financ Rev.

