# Supplementary material for: Association Between Long‑Term Exposure to Air Pollution and the Rate of Mortality After Hip Fracture Surgery in Patients Older Than 60 Years: Nationwide Cohort Study in Taiwan
Source: JMIR Public Health Surveill. 2024 Mar 18;10:e46591. doi: 10.2196/46591 (PMC10985614; doi:10.2196/46591)
Supplement: Multimedia Appendix 1 [file publichealth_v10i1e46591_app1.docx]

## Multimedia Appendix 1. The International Classification of Diseases, Ninth Revision, (ICD-9) codes and Anatomical Therapeutic Chemical (ATC) Classification code established in the inclusion criteria, exclusion criteria, and definition of confounders.

### Inclusion criteria: those underwent hip fracture surgery between January 1, 2000 and December 31, 2012 based on:

1. International Classification of Disease, Ninth Revision, Clinical Modification (ICD-9-CM) codes 820, 820.0, 820.00, 820.01, 820.02, 820.09, 820.8, 820.03, 820.2, 820.20
2. ICD-9-CM codes 79.15, 79.35, 81.52

### Exclusion criteria

1. unreasonable age (0 years old)
2. age of <60 years at baseline; with the outcome diagnosis prior to the start of the study (to prevent reverse causation bias)
3. history of pathological fracture (ICD-9-CM codes 733.14 and 733.15), open fracture (ICD-9-CM codes 820.1, 820.10, 820.11, 820.12, 820.19, 820.9, 820.13, 820.22, 820.3, 820.30, 820.31, and 820.32), or major traffic accident (ICD-9 code: E810-E819, E881-E883, E884.1) before the initiation of the study
4. whose follow-up start date was the same as the follow-up end date
5. subjects were excluded if their survival date were before 2003-07-01

## Definition of confounders

1. Hip fracture procedure

(ICD-9-CM codes 79.15, 79.35, 81.52)

1. Co-medications

(cumulative use of medications other than anti-osteoporosis drugs for more than 90 days)

1. Anti-osteoporosis medication

Alendronate:

(ATC codes A049787100, AC49787100, AC57366100, B022015100, B023167100, B024168100, B024480100, B024588100, B025104100, B025364100, B025890100, B026136100, BB25104100)

Risedronate:
(ATC codes B023887100, B025845100, B026261100, BA25845100, BC26272100, BC27022100)

Ibandronate:

(ATC codes B024630216, AC57913216, BC24630216)

Zoledronic:

(ATC codes B024692255, BC24692255)

Denosumab

(ATC codes K000918209, KC00918209)

Raloxifene

(ATC codes B022717100, B024023100, B025807100, B026236100, AC59410100, BB24023100, BC24023100, BC25807100, BC26236100)
