# Supplementary material for: Association Between Long‑Term Exposure to Air Pollution and the Rate of Mortality After Hip Fracture Surgery in Patients Older Than 60 Years: Nationwide Cohort Study in Taiwan
Source: JMIR Public Health Surveill. 2024 Mar 18;10:e46591. doi: 10.2196/46591 (PMC10985614; doi:10.2196/46591)
Supplement: Multimedia Appendix 2 [file publichealth_v10i1e46591_app2.docx]

## Multimedia Appendix 2. Characteristics of the study population across the tertiles of SO_2_ exposure.

| **Characteristics** | **Tertiles^a^ of average daily SO_2_^b^, n (%)** | | | ***P* value** | **Total (N = 7426)** |
| --- | --- | --- | --- | --- | --- |
|  | **T1 (lowest) (n = 2391)** | **T2 (n = 2541)** | **T3 (highest) (n = 2494)** |  |  |
| **Death** | 229 (9.58) | 264 (10.39) | 436 (17.48) | <.001 | 929 (12.51) |
| **Men** | 910 (38.06) | 906 (35.66) | 1110 (44.51) | <.001 | 2926 (39.40) |
| **Age (years)** | | | | .778 |  |
| 60 to 79 | 1251 (52.32) | 1331 (52.38) | 1284 (51.48) |  | 3866 (52.06) |
| ≥80 | 1140 (47.68) | 1210 (47.62) | 1210 (48.52) |  | 3560 (47.94) |
| Mean ± SD^c^ | 78.51 ± 7.96 | 78.36 ± 8.31 | 78.75 ± 7.93 | .222 | 78.54 ± 8.07 |
| **Urbanization level** | | | | <.001 |  |
| 1 (highest) | 1029 (43.04) | 1219 (47.97) | 1024 (41.06) |  | 3272 (44.06) |
| 2 | 1063 (44.46) | 789 (31.05) | 913 (36.61) |  | 2765 (37.23) |
| 3 | 143 (5.98) | 291 (11.45) | 277 (11.11) |  | 711 (9.57) |
| 4 (lowest) | 1 (.04) | 42 (1.65) | 69 (2.77) |  | 112 (1.51) |
| Unknown | 155 (6.48) | 200 (7.87) | 211 (8.46) |  | 566 (7.62) |
| **Insurance amount (New Taiwan Dollar)** | | | | <.001 |  |
| Financially dependent | 6 (.25) | 12 (.47) | 6 (.24) |  | 24 (.32) |
| 1 to 19 999 | 976 (40.82) | 1171 (46.08) | 1390 (55.73) |  | 3537 (47.63) |
| 20 000 to 39 999 | 1044 (43.66) | 761 (29.95) | 568 (22.77) |  | 2373 (31.96) |
| ≥40 000 | 33 (1.38) | 51 (2.01) | 35 (1.40) |  | 119 (1.60) |
| Unknown | 332 (13.89) | 546 (21.49) | 495 (19.85) |  | 1373 (18.49) |
| **CCI^d^ score (mean ± SD^c^)** | 4.46 ± 2.94 | 4.57 ± 2.99 | 4.67 ± 2.98 | .046 | 4.57 ± 2.97 |
| **Hip fracture procedure** | | | | .312 |  |
| Closed reduction of fracture with internal fixation | 141 (5.90) | 169 (6.65) | 138 (5.53) |  | 448 (6.03) |
| Open reduction of fracture with internal fixation | 1283 (53.66) | 1319 (51.91) | 1355 (54.33) |  | 3957 (53.29) |
| Partial hip replacement | 967 (40.44) | 1053 (41.44) | 1001 (40.14) |  | 3021 (40.68) |
| **Co-medications** | 2030 (84.90) | 2175 (85.60) | 2139 (85.77) | .664 | 6344 (85.43) |
| **Anti-osteoporosis medication** | | | |  |  |
| Alendronate | 268 (11.21) | 281 (11.06) | 203 (8.14) | <.001 | 752 (10.13) |
| Risedronate | 0 (0.00) | 0 (0.00) | 0 (0.00) | - | 0 (0.00) |
| Ibandronate | 7 (0.29) | 2 (0.08) | 2 (0.08) | .108 | 11 (0.15) |
| Zoledronic | 0 (0.00) | 0 (0.00) | 0 (0.00) | - | 0 (0.00) |
| Denosumab | 0 (0.00) | 0 (0.00) | 0 (0.00) | - | 0 (0.00) |
| Raloxifene | 69 (2.89) | 90 (3.54) | 77 (3.09) | .402 | 236 (3.18) |
| ^a^The tertile values, in ppb, were as follows: T1: < 3.59; T2: >= 3.59 and < 4.06; T3: >= 4.06.  ^b^SO_2_: sulfur dioxide.  ^c^SD: standard deviation.  ^d^CCI score: Charlson Comorbidity Index score. | | | | | |
