# Supplementary material for: Association Between Long‑Term Exposure to Air Pollution and the Rate of Mortality After Hip Fracture Surgery in Patients Older Than 60 Years: Nationwide Cohort Study in Taiwan
Source: JMIR Public Health Surveill. 2024 Mar 18;10:e46591. doi: 10.2196/46591 (PMC10985614; doi:10.2196/46591)
Supplement: Multimedia Appendix 3 [file publichealth_v10i1e46591_app3.docx]

## Multimedia Appendix 3. Characteristics of the study population across the tertiles of CO_2_ exposure.

| **Characteristics** | **Tertiles^a^ of average daily CO_2_^b^, n (%)** | | | ***P* value** | **Total (N = 3812)** |
| --- | --- | --- | --- | --- | --- |
|  | **T1 (lowest)**  **(n = 1271)** | **T2 (n = 1269)** | **T3 (highest) (n = 1272)** |  |  |
| **Death** | 252 (19.83) | 86 (6.78) | 64 (5.03) | <.001 | 402 (10.55) |
| **Men** | 501 (39.42) | 461 (36.33) | 418 (32.86) | .003 | 1380 (36.20) |
| **Age (years)** | | | | <.001 |  |
| 60 to 79 | 614 (48.31) | 686 (54.06) | 722 (56.76) |  | 2022 (53.04) |
| ≥80 | 657 (51.69) | 583 (45.94) | 550 (43.24) |  | 1790 (46.96) |
| Mean ± SD^c^ | 79.24 ± 8.00 | 78.00 ± 8.46 | 77.43 ± 8.31 | <.001 | 78.22 ± 8.29 |
| **Urbanization level** | | | | <.001 |  |
| 1 (highest) | 558 (43.90) | 685 (53.98) | 488 (38.36) |  | 1731 (45.41) |
| 2 | 406 (31.94) | 391 (30.81) | 510 (40.09) |  | 1307 (34.29) |
| 3 | 148 (11.64) | 60 (4.73) | 183 (14.39) |  | 391 (10.26) |
| 4 (lowest) | 10 (.79) | 16 (1.26) | 4 (.31) |  | 30 (.79) |
| Unknown | 149 (11.72) | 117 (9.22) | 87 (6.84) |  | 353 (9.26) |
| **Insurance amount (New Taiwan Dollar)** | | | | <.001 |  |
| Financially dependent | 1 (.08) | 5 (.39) | 6 (.47) |  | 12 (.31) |
| 1 to 19 999 | 719 (56.57) | 664 (52.32) | 486 (38.21) |  | 1869 (49.03) |
| 20 000 to 39 999 | 229 (18.02) | 233 (18.36) | 527 (41.43) |  | 989 (25.94) |
| ≥40 000 | 19 (1.49) | 34 (2.68) | 35 (2.75) |  | 88 (2.31) |
| Unknown | 303 (23.84) | 333 (26.24) | 218 (17.14) |  | 854 (22.40) |
| **CCI^d^ score (mean ± SD^c^)** | 4.88 ± 2.96 | 4.50 ± 3.01 | 4.18 ± 2.81 | <.001 | 4.52 ± 2.94 |
| **Hip fracture procedure** | | | | .008 |  |
| Closed reduction of fracture with internal fixation | 62 (4.88) | 97 (7.64) | 81 (6.37) |  | 240 (6.30) |
| Open reduction of fracture with internal fixation | 702 (55.23) | 626 (49.33) | 674 (52.99) |  | 2002 (52.52) |
| Partial hip replacement | 507 (39.89) | 546 (43.03) | 517 (40.64) |  | 1570 (41.19) |
| **Co-medications** | 1103 (86.78) | 1071 (84.40) | 1066 (83.81) | .084 | 3240 (84.99) |
| **Anti-osteoporosis medication** | | | |  |  |
| Alendronate | 83 (6.53) | 144 (11.35) | 166 (13.05) | <.001 | 393 (10.31) |
| Risedronate | 0 (0.00) | 0 (0.00) | 0 (0.00) | - | 0 (0.00) |
| Ibandronate | 1 (0.08) | 2 (0.16) | 2 (0.16) | .876 | 5 (0.13) |
| Zoledronic | 0 (0.00) | 0 (0.00) | 0 (0.00) | - | 0 (0.00) |
| Denosumab | 0 (0.00) | 0 (0.00) | 0 (0.00) | - | 0 (0.00) |
| Raloxifene | 36 (2.83) | 51 (4.02) | 43 (3.38) | .257 | 130 (3.41) |
| ^a^The tertile values, in ppm, were as follows: T1: < 393.97; T2: >= 393.97 and < 401.51; T3: >= 401.51.  ^b^CO_2_: carbon dioxide.  ^c^SD: standard deviation.  ^d^CCI score: Charlson Comorbidity Index score. | | | | | |
