# Supplementary material for: Association Between Long‑Term Exposure to Air Pollution and the Rate of Mortality After Hip Fracture Surgery in Patients Older Than 60 Years: Nationwide Cohort Study in Taiwan
Source: JMIR Public Health Surveill. 2024 Mar 18;10:e46591. doi: 10.2196/46591 (PMC10985614; doi:10.2196/46591)
Supplement: Multimedia Appendix 4 [file publichealth_v10i1e46591_app4.docx]

## Multimedia Appendix 4. Characteristics of the study population across the tertiles of CO exposure.

| **Characteristics** | **Tertiles^a^ of average daily CO^b^, n (%)** | | | ***P* value** | **Total (N = 7426)** |
| --- | --- | --- | --- | --- | --- |
|  | **T1 (lowest) (n = 2438)** | **T2 (n = 2512)** | **T3 (highest) (n = 2476)** |  |  |
| **Death** | 180 (7.38) | 300 (11.94) | 449 (18.13) | <.001 | 929 (12.51) |
| **Men** | 867 (35.56) | 983 (39.13) | 1076 (43.46) | <.001 | 2926 (39.40) |
| **Age (years)** | | | | <.001 |  |
| 60 to 79 | 1336 (54.80) | 1349 (53.70) | 1181 (47.70) |  | 3866 (52.06) |
| ≥80 | 1102 (45.20) | 1163 (46.30) | 1295 (52.30) |  | 3560 (47.94) |
| Mean ± SD^c^ | 78.09 ± 8.06 | 78.25 ± 8.01 | 79.27 ± 8.11 | <.001 | 78.54 ± 8.07 |
| **Urbanization level** | | | | <.001 |  |
| 1 (highest) | 908 (37.24) | 1102 (43.87) | 1262 (50.97) |  | 3272 (44.06) |
| 2 | 1029 (42.21) | 977 (38.89) | 759 (30.65) |  | 2765 (37.23) |
| 3 | 345 (14.15) | 189 (7.52) | 177 (7.15) |  | 711 (9.57) |
| 4 (lowest) | 5 (.21) | 43 (1.71) | 64 (2.58) |  | 112 (1.51) |
| Unknown | 151 (6.19) | 201 (8.00) | 214 (8.64) |  | 566 (7.62) |
| **Insurance amount (New Taiwan Dollar)** | | | | <.001 |  |
| Financially dependent | 10 (.41) | 8 (.32) | 6 (.24) |  | 24 (.32) |
| 1 to 19 999 | 729 (29.90) | 1288 (51.27) | 1520 (61.39) |  | 3537 (47.63) |
| 20 000 to 39 999 | 1417 (58.12) | 691 (27.51) | 265 (10.70) |  | 2373 (31.96) |
| ≥40 000 | 33 (1.35) | 46 (1.83) | 40 (1.62) |  | 119 (1.60) |
| Unknown | 249 (10.21) | 479 (19.07) | 645 (26.05) |  | 1373 (18.49) |
| **CCI^d^ score (mean ± SD^c^)** | 4.40 ± 2.89 | 4.64 ± 2.99 | 4.67 ± 3.02 | .002 | 4.57 ± 2.97 |
| **Hip fracture procedure** | | | | .125 |  |
| Closed reduction of fracture with internal fixation | 143 (5.87) | 136 (5.41) | 169 (6.83) |  | 448 (6.03) |
| Open reduction of fracture with internal fixation | 1332 (54.63) | 1339 (53.30) | 1286 (51.94) |  | 3957 (53.29) |
| Partial hip replacement | 963 (39.50) | 1037 (41.28) | 1021 (41.24) |  | 3021 (40.68) |
| **Co-medications** | 2095 (85.93) | 2160 (85.99) | 2089 (84.37) | .187 | 6344 (85.43) |
| **Anti-osteoporosis medication** | | | |  |  |
| Alendronate | 312 (12.80) | 232 (9.24) | 208 (8.40) | <.001 | 752 (10.13) |
| Risedronate | 0 (0.00) | 0 (0.00) | 0 (0.00) | - | 0 (0.00) |
| Ibandronate | 6 (0.25) | 4 (0.16) | 1 (0.04) | .169 | 11 (0.15) |
| Zoledronic | 0 (0.00) | 0 (0.00) | 0 (0.00) | - | 0 (0.00) |
| Denosumab | 0 (0.00) | 0 (0.00) | 0 (0.00) | - | 0 (0.00) |
| Raloxifene | 87 (3.57) | 78 (3.11) | 71 (2.87) | .363 | 236 (3.18) |
| ^a^The tertile values, in ppm, were as follows: T1: < .47; T2: >= .47 and < .61; T3: >= .61.  ^b^CO: carbon monoxide.  ^c^SD: standard deviation.  ^d^CCI score: Charlson Comorbidity Index score. | | | | | |
