# Supplementary material for: Association Between Long‑Term Exposure to Air Pollution and the Rate of Mortality After Hip Fracture Surgery in Patients Older Than 60 Years: Nationwide Cohort Study in Taiwan
Source: JMIR Public Health Surveill. 2024 Mar 18;10:e46591. doi: 10.2196/46591 (PMC10985614; doi:10.2196/46591)
Supplement: Multimedia Appendix 5 [file publichealth_v10i1e46591_app5.docx]

## Multimedia Appendix 5. Characteristics of the study population across the tertiles of O_3_ exposure.

| **Characteristics** | **Tertiles^a^ of average daily O_3_^b^, n (%)** | | | ***P* value** | **Total (N = 7426)** |
| --- | --- | --- | --- | --- | --- |
|  | **T1 (lowest) (n = 2343)** | **T2 (n = 2607)** | **T3 (highest) (n = 2476)** |  |  |
| **Death** | 574 (24.50) | 178 (6.83) | 177 (7.15) | <.001 | 929 (12.51) |
| **Men** | 1050 (44.81) | 1015 (38.93) | 861 (34.77) | <.001 | 2926 (39.40) |
| **Age (years)** | | | | <.001 |  |
| 60 to 79 | 1062 (45.33) | 1410 (54.09) | 1394 (56.30) |  | 3866 (52.06) |
| ≥80 | 1281 (54.67) | 1197 (45.91) | 1082 (43.70) |  | 3560 (47.94) |
| Mean ± SD^c^ | 79.94 ± 7.78 | 77.88 ± 8.17 | 77.91 ± 8.08 | <.001 | 78.54 ± 8.07 |
| **Urbanization level** | | | | <.001 |  |
| 1 (highest) | 1062 (45.33) | 1261 (48.37) | 949 (38.33) |  | 3272 (44.06) |
| 2 | 878 (37.47) | 906 (34.75) | 981 (39.62) |  | 2765 (37.23) |
| 3 | 174 (7.43) | 227 (8.71) | 310 (12.52) |  | 711 (9.57) |
| 4 (lowest) | 29 (1.24) | 40 (1.53) | 43 (1.74) |  | 112 (1.51) |
| Unknown | 200 (8.54) | 173 (6.64) | 193 (7.79) |  | 566 (7.62) |
| **Insurance amount (New Taiwan Dollar)** | | | | <.001 |  |
| Financially dependent | 5 (.21) | 11 (.42) | 8 (.32) |  | 24 (.32) |
| 1 to 19 999 | 1415 (60.39) | 1274 (48.87) | 848 (34.25) |  | 3537 (47.63) |
| 20 000 to 39 999 | 384 (16.39) | 768 (29.46) | 1221 (49.31) |  | 2373 (31.96) |
| ≥40 000 | 24 (1.02) | 52 (1.99) | 43 (1.74) |  | 119 (1.60) |
| Unknown | 515 (21.98) | 502 (19.26) | 356 (14.38) |  | 1373 (18.49) |
| **CCI^d^ score (mean ± SD^c^)** | 4.92 ± 3.00 | 4.43 ± 2.99 | 4.39 ± 2.89 | <.001 | 4.57 ± 2.97 |
| **Hip fracture procedure** | | | | .049 |  |
| Closed reduction of fracture with internal fixation | 129 (5.51) | 170 (6.52) | 149 (6.02) |  | 448 (6.03) |
| Open reduction of fracture with internal fixation | 1306 (55.74) | 1367 (52.44) | 1284 (51.86) |  | 3957 (53.29) |
| Partial hip replacement | 908 (38.75) | 1070 (41.04) | 1043 (42.12) |  | 3021 (40.68) |
| **Co-medications** | 2005 (85.57) | 2211 (84.81) | 2128 (85.95) | .504 | 6344 (85.43) |
| **Anti-osteoporosis medication** | | | |  |  |
| Alendronate | 148 (6.32) | 320 (12.27) | 284 (11.47) | <.001 | 752 (10.13) |
| Risedronate | 0 (0.00) | 0 (0.00) | 0 (0.00) | - | 0 (0.00) |
| Ibandronate | 0 (0.00) | 5 (0.19) | 6 (0.24) | .043 | 11 (0.15) |
| Zoledronic | 0 (0.00) | 0 (0.00) | 0 (0.00) | - | 0 (0.00) |
| Denosumab | 0 (0.00) | 0 (0.00) | 0 (0.00) | - | 0 (0.00) |
| Raloxifene | 58 (2.48) | 101 (3.87) | 77 (3.11) | .019 | 236 (3.18) |
| ^a^The tertile values, in ppb, were as follows: T1: < 27.31; T2: >= 27.31 and < 28.83; T3: >= 28.83.  ^b^O_3_: ozone.  ^c^SD: standard deviation.  ^d^CCI score: Charlson Comorbidity Index score. | | | | | |
