# Supplementary material for: Association Between Long‑Term Exposure to Air Pollution and the Rate of Mortality After Hip Fracture Surgery in Patients Older Than 60 Years: Nationwide Cohort Study in Taiwan
Source: JMIR Public Health Surveill. 2024 Mar 18;10:e46591. doi: 10.2196/46591 (PMC10985614; doi:10.2196/46591)
Supplement: Multimedia Appendix 6 [file publichealth_v10i1e46591_app6.docx]

## Multimedia Appendix 6. Characteristics of the study population across the tertiles of PM_10_ exposure.

| **Characteristics** | **Tertiles^a^ of average daily PM_10_^b^, n (%)** | | | ***P* value** | **Total (N = 7426)** |
| --- | --- | --- | --- | --- | --- |
|  | **T1 (lowest) (n = 2475)** | **T2 (n = 2335)** | **T3 (highest) (n = 2616)** |  |  |
| **Death** | 274 (11.07) | 266 (11.39) | 389 (14.87) | <.001 | 929 (12.51) |
| **Men** | 984 (39.76) | 893 (38.24) | 1049 (40.10) | .372 | 2926 (39.40) |
| **Age (years)** | | | | .002 |  |
| 60 to 79 | 1218 (49.21) | 1236 (52.93) | 1412 (53.98) |  | 3866 (52.06) |
| ≥80 | 1257 (50.79) | 1099 (47.07) | 1204 (46.02) |  | 3560 (47.94) |
| Mean ± SD^c^ | 78.95 ± 8.18 | 78.31 ± 8.13 | 78.36 ± 7.90 | .008 | 78.54 ± 8.07 |
| **Urbanization level** | | | | <.001 |  |
| 1 (highest) | 1320 (53.33) | 1011 (43.30) | 941 (35.97) |  | 3272 (44.06) |
| 2 | 795 (32.12) | 858 (36.75) | 1112 (42.51) |  | 2765 (37.23) |
| 3 | 151 (6.10) | 233 (9.98) | 327 (12.50) |  | 711 (9.57) |
| 4 (lowest) | 45 (1.82) | 20 (.86) | 47 (1.80) |  | 112 (1.51) |
| Unknown | 164 (6.63) | 213 (9.12) | 189 (7.22) |  | 566 (7.62) |
| **Insurance amount (New Taiwan Dollar)** | | | | <.001 |  |
| Financially dependent | 8 (.32) | 7 (.30) | 9 (.34) |  | 24 (.32) |
| 1 to 19 999 | 1278 (51.64) | 1126 (48.22) | 1133 (43.31) |  | 3537 (47.63) |
| 20 000 to 39 999 | 639 (25.82) | 675 (28.91) | 1059 (40.48) |  | 2373 (31.96) |
| ≥40 000 | 41 (1.66) | 51 (2.18) | 27 (1.03) |  | 119 (1.60) |
| Unknown | 509 (20.57) | 476 (20.39) | 388 (14.83) |  | 1373 (18.49) |
| **CCI^d^ score (mean ± SD^c^)** | 4.52 ± 3.01 | 4.49 ± 2.89 | 4.69 ± 3.00 | .029 | 4.57 ± 2.97 |
| **Hip fracture procedure** | | | | .346 |  |
| Closed reduction of fracture with internal fixation | 132 (5.33) | 156 (6.68) | 160 (6.12) |  | 448 (6.03) |
| Open reduction of fracture with internal fixation | 1343 (54.26) | 1232 (52.76) | 1382 (52.83) |  | 3957 (53.29) |
| Partial hip replacement | 1000 (40.40) | 947 (40.56) | 1074 (41.06) |  | 3021 (40.68) |
| **Co-medications** | 2090 (84.44) | 1992 (85.31) | 2262 (86.47) | .121 | 6344 (85.43) |
| **Anti-osteoporosis medication** | | | |  |  |
| Alendronate | 274 (11.07) | 206 (8.82) | 272 (10.40) | .030 | 752 (10.13) |
| Risedronate | 0 (0.00) | 0 (0.00) | 0 (0.00) | - | 0 (0.00) |
| Ibandronate | 3 (0.12) | 2 (0.09) | 6 (0.23) | .417 | 11 (0.15) |
| Zoledronic | 0 (0.00) | 0 (0.00) | 0 (0.00) | - | 0 (0.00) |
| Denosumab | 0 (0.00) | 0 (0.00) | 0 (0.00) | - | 0 (0.00) |
| Raloxifene | 69 (2.79) | 75 (3.21) | 92 (3.52) | .331 | 236 (3.18) |
| ^a^The tertile values, in μg/m^3^, were as follows: T1: < 50.25; T2: >= 50.25 and < 58.45; T3: >= 58.45.  ^b^PM_10_: particulate matters having a size of <10 μm.  ^c^SD: standard deviation.  ^d^CCI score: Charlson Comorbidity Index score. | | | | | |
