# Supplementary material for: Association Between Long‑Term Exposure to Air Pollution and the Rate of Mortality After Hip Fracture Surgery in Patients Older Than 60 Years: Nationwide Cohort Study in Taiwan
Source: JMIR Public Health Surveill. 2024 Mar 18;10:e46591. doi: 10.2196/46591 (PMC10985614; doi:10.2196/46591)
Supplement: Multimedia Appendix 7 [file publichealth_v10i1e46591_app7.docx]

## Multimedia Appendix 7. Characteristics of the study population across the tertiles of PM_2.5_ exposure.

| **Characteristics** | **Tertiles^a^ of average daily PM_2.5_^b^, n (%)** | | | ***P* value** | **Total (N = 7252)** |
| --- | --- | --- | --- | --- | --- |
|  | **T1 (lowest) (n = 2417)** | **T2 (n = 2417)** | **T3 (highest) (n = 2418)** |  |  |
| **Death** | 240 (9.93) | 245 (10.14) | 361 (14.93) | <.001 | 846 (11.67) |
| **Men** | 965 (39.93) | 883 (36.53) | 998 (41.27) | .002 | 2846 (39.24) |
| **Age (years)** | | | | .009 |  |
| 60 to 79 | 1206 (49.90) | 1280 (52.96) | 1310 (54.18) |  | 3796 (52.34) |
| ≥80 | 1211 (50.10) | 1137 (47.04) | 1108 (45.82) |  | 3456 (47.66) |
| Mean ± SD^c^ | 78.80 ± 8.09 | 78.23 ± 8.29 | 78.41 ± 7.85 | .042 | 78.48 ± 8.08 |
| **Urbanization level** | | | | <.001 |  |
| 1 (highest) | 1243 (51.43) | 1066 (44.10) | 897 (37.10) |  | 3206 (44.21) |
| 2 | 865 (35.79) | 820 (33.93) | 1002 (41.44) |  | 2687 (37.05) |
| 3 | 118 (4.88) | 311 (12.87) | 266 (11.00) |  | 695 (9.58) |
| 4 (lowest) | 35 (1.45) | 24 (.99) | 53 (2.19) |  | 112 (1.54) |
| Unknown | 156 (6.45) | 196 (8.11) | 200 (8.27) |  | 552 (7.61) |
| **Insurance amount (New Taiwan Dollar)** | | | | <.001 |  |
| Financially dependent | 8 (.33) | 8 (.33) | 8 (.33) |  | 24 (.33) |
| 1 to 19 999 | 1236 (51.14) | 1020 (42.20) | 1141 (47.19) |  | 3397 (46.84) |
| 20 000 to 39 999 | 656 (27.14) | 889 (36.78) | 825 (34.12) |  | 2370 (32.68) |
| ≥40 000 | 40 (1.65) | 50 (2.07) | 29 (1.20) |  | 119 (1.64) |
| Unknown | 477 (19.74) | 450 (18.62) | 415 (17.16) |  | 1342 (18.51) |
| **CCI^d^ score (mean ± SD^c^)** | 4.47 ± 2.99 | 4.52 ± 2.94 | 4.71 ± 2.98 | .012 | 4.56 ± 2.97 |
| **Hip fracture procedure** | | | | .493 |  |
| Closed reduction of fracture with internal fixation | 135 (5.59) | 144 (5.96) | 162 (6.70) |  | 441 (6.08) |
| Open reduction of fracture with internal fixation | 1309 (54.16) | 1293 (53.50) | 1267 (52.40) |  | 3869 (53.35) |
| Partial hip replacement | 973 (40.26) | 980 (40.55) | 989 (40.90) |  | 2942 (40.57) |
| **Co-medications** | 2026 (83.82) | 2084 (86.22) | 2094 (86.60) | .012 | 6204 (85.55) |
| **Anti-osteoporosis medication** | | | |  |  |
| Alendronate | 266 (11.01) | 253 (10.47) | 231 (9.55) | .245 | 750 (10.34) |
| Risedronate | 0 (0.00) | 0 (0.00) | 0 (0.00) | - | 0 (0.00) |
| Ibandronate | 1 (0.04) | 5 (0.21) | 5 (0.21) | .256 | 11 (0.15) |
| Zoledronic | 0 (0.00) | 0 (0.00) | 0 (0.00) | - | 0 (0.00) |
| Denosumab | 0 (0.00) | 0 (0.00) | 0 (0.00) | - | 0 (0.00) |
| Raloxifene | 69 (2.85) | 92 (3.81) | 75 (3.10) | .154 | 236 (3.25) |
| ^a^The tertile values, in μg/m^3^, were as follows: T1: < 29.98; T2: >= 29.98 and < 36.49; T3: >= 36.49.  ^b^PM_2.5_: particulate matters having a size of <2.5 μm.  ^c^SD: standard deviation.  ^d^CCI score: Charlson Comorbidity Index score. | | | | | |
