# Supplementary material for: Association Between Long‑Term Exposure to Air Pollution and the Rate of Mortality After Hip Fracture Surgery in Patients Older Than 60 Years: Nationwide Cohort Study in Taiwan
Source: JMIR Public Health Surveill. 2024 Mar 18;10:e46591. doi: 10.2196/46591 (PMC10985614; doi:10.2196/46591)
Supplement: Multimedia Appendix 8 [file publichealth_v10i1e46591_app8.docx]

## Multimedia Appendix 8. Characteristics of the study population across the tertiles of NO_X_ exposure.

| **Characteristics** | **Tertiles^a^ of average daily NO_X_^b^, n (%)** | | | ***P* value** | **Total (N = 7426)** |
| --- | --- | --- | --- | --- | --- |
|  | **T1 (lowest) (n = 2439)** | **T2 (n = 2511)** | **T3 (highest) (n = 2476)** |  |  |
| **Death** | 199 (8.16) | 329 (13.10) | 401 (16.20) | <.001 | 929 (12.51) |
| **Men** | 877 (35.96) | 978 (38.95) | 1071 (43.26) | <.001 | 2926 (39.40) |
| **Age (years)** | | | | <.001 |  |
| 60 to 79 | 1330 (54.53) | 1331 (53.01) | 1205 (48.67) |  | 3866 (52.06) |
| ≥80 | 1109 (45.47) | 1180 (46.99) | 1271 (51.33) |  | 3560 (47.94) |
| Mean ± SD^c^ | 78.17 ± 8.02 | 78.39 ± 8.05 | 79.05 ± 8.12 | <.001 | 78.54 ± 8.07 |
| **Urbanization level** | | | | <.001 |  |
| 1 (highest) | 888 (36.41) | 1087 (43.29) | 1297 (52.38) |  | 3272 (44.06) |
| 2 | 1084 (44.44) | 919 (36.60) | 762 (30.78) |  | 2765 (37.23) |
| 3 | 305 (12.51) | 248 (9.88) | 158 (6.38) |  | 711 (9.57) |
| 4 (lowest) | 1 (.04) | 45 (1.79) | 66 (2.67) |  | 112 (1.51) |
| Unknown | 161 (6.60) | 212 (8.44) | 193 (7.79) |  | 566 (7.62) |
| **Insurance amount (New Taiwan Dollar)** | | | | <.001 |  |
| Financially dependent | 8 (.33) | 10 (.40) | 6 (.24) |  | 24 (.32) |
| 1 to 19 999 | 767 (31.45) | 1270 (50.58) | 1500 (60.58) |  | 3537 (47.63) |
| 20 000 to 39 999 | 1408 (57.73) | 692 (27.56) | 273 (11.03) |  | 2373 (31.96) |
| ≥40 000 | 32 (1.31) | 40 (1.59) | 47 (1.90) |  | 119 (1.60) |
| Unknown | 224 (9.18) | 499 (19.87) | 650 (26.25) |  | 1373 (18.49) |
| **CCI^d^ score (mean ± SD^c^)** | 4.40 ± 2.88 | 4.66 ± 3.01 | 4.64 ± 3.01 | .002 | 4.57 ± 2.97 |
| **Hip fracture procedure** | | | | .025 |  |
| Closed reduction of fracture with internal fixation | 130 (5.33) | 143 (5.69) | 175 (7.07) |  | 448 (6.03) |
| Open reduction of fracture with internal fixation | 1332 (54.61) | 1358 (54.08) | 1267 (51.17) |  | 3957 (53.29) |
| Partial hip replacement | 977 (40.06) | 1010 (40.22) | 1034 (41.76) |  | 3021 (40.68) |
| **Co-medications** | 2089 (85.65) | 2169 (86.38) | 2086 (84.25) | .096 | 6344 (85.43) |
| **Anti-osteoporosis medication** | | | |  |  |
| Alendronate | 294 (12.05) | 228 (9.08) | 230 (9.29) | <.001 | 752 (10.13) |
| Risedronate | 0 (0.00) | 0 (0.00) | 0 (0.00) | - | 0 (0.00) |
| Ibandronate | 4 (0.16) | 5 (0.20) | 2 (0.08) | .567 | 11 (0.15) |
| Zoledronic | 0 (0.00) | 0 (0.00) | 0 (0.00) | - | 0 (0.00) |
| Denosumab | 0 (0.00) | 0 (0.00) | 0 (0.00) | - | 0 (0.00) |
| Raloxifene | 80 (3.28) | 81 (3.23) | 75 (3.03) | .870 | 236 (3.18) |
| ^a^The tertile values, in ppb, were as follows: T1: < 20.59; T2: >= 20.59 and < 28.32; T3: >= 28.32.  ^b^NO_X_: nitrogen oxides.  ^c^SD: standard deviation.  ^d^CCI score: Charlson Comorbidity Index score. | | | | | |
