# Supplementary material for: Association Between Long‑Term Exposure to Air Pollution and the Rate of Mortality After Hip Fracture Surgery in Patients Older Than 60 Years: Nationwide Cohort Study in Taiwan
Source: JMIR Public Health Surveill. 2024 Mar 18;10:e46591. doi: 10.2196/46591 (PMC10985614; doi:10.2196/46591)
Supplement: Multimedia Appendix 9 [file publichealth_v10i1e46591_app9.docx]

## Multimedia Appendix 9. Characteristics of the study population across the tertiles of NO exposure.

| **Characteristics** | **Tertiles^a^ of average daily NO^b^, n (%)** | | | ***P* value** | **Total (N = 7426)** |
| --- | --- | --- | --- | --- | --- |
|  | **T1 (lowest) (n = 2409)** | **T2 (n = 2541)** | **T3 (highest) (n = 2476)** |  |  |
| **Death** | 215 (8.92) | 325 (12.79) | 389 (15.71) | <.001 | 929 (12.51) |
| **Men** | 887 (36.82) | 994 (39.12) | 1045 (42.21) | <.001 | 2926 (39.40) |
| **Age (years)** | | | | <.001 |  |
| 60 to 79 | 1328 (55.13) | 1344 (52.89) | 1194 (48.22) |  | 3866 (52.06) |
| ≥80 | 1081 (44.87) | 1197 (47.11) | 1282 (51.78) |  | 3560 (47.94) |
| Mean ± SD^c^ | 78.10 ± 7.95 | 78.39 ± 8.09 | 79.12 ± 8.14 | <.001 | 78.54 ± 8.07 |
| **Urbanization level** | | | | <.001 |  |
| 1 (highest) | 880 (36.53) | 1059 (41.68) | 1333 (53.84) |  | 3272 (44.06) |
| 2 | 1064 (44.17) | 991 (39.00) | 710 (28.68) |  | 2765 (37.23) |
| 3 | 321 (13.33) | 231 (9.09) | 159 (6.42) |  | 711 (9.57) |
| 4 (lowest) | 1 (.04) | 45 (1.77) | 66 (2.67) |  | 112 (1.51) |
| Unknown | 143 (5.94) | 215 (8.46) | 208 (8.40) |  | 566 (7.62) |
| **Insurance amount (New Taiwan Dollar)** | | | | <.001 |  |
| Financially dependent | 8 (.33) | 11 (.43) | 5 (.20) |  | 24 (.32) |
| 1 to 19 999 | 758 (31.47) | 1290 (50.77) | 1489 (60.14) |  | 3537 (47.63) |
| 20 000 to 39 999 | 1403 (58.24) | 699 (27.51) | 271 (10.95) |  | 2373 (31.96) |
| ≥40 000 | 26 (1.08) | 46 (1.81) | 47 (1.90) |  | 119 (1.60) |
| Unknown | 214 (8.88) | 495 (19.48) | 664 (26.82) |  | 1373 (18.49) |
| **CCI^d^ score (mean ± SD^c^)** | 4.41 ± 2.90 | 4.64 ± 2.99 | 4.65 ± 3.01 | .006 | 4.57 ± 2.97 |
| **Hip fracture procedure** | | | | .017 |  |
| Closed reduction of fracture with internal fixation | 126 (5.23) | 148 (5.82) | 174 (7.03) |  | 448 (6.03) |
| Open reduction of fracture with internal fixation | 1327 (55.09) | 1365 (53.72) | 1265 (51.09) |  | 3957 (53.29) |
| Partial hip replacement | 956 (39.68) | 1028 (40.46) | 1037 (41.88) |  | 3021 (40.68) |
| **Co-medications** | 2068 (85.84) | 2192 (86.27) | 2084 (84.17) | .085 | 6344 (85.43) |
| **Anti-osteoporosis medication** | | | |  |  |
| Alendronate | 303 (12.58) | 221 (8.70) | 228 (9.21) | <.001 | 752 (10.13) |
| Risedronate | 0 (0.00) | 0 (0.00) | 0 (0.00) | - | 0 (0.00) |
| Ibandronate | 6 (0.25) | 2 (0.08) | 3 (0.12) | .280 | 11 (0.15) |
| Zoledronic | 0 (0.00) | 0 (0.00) | 0 (0.00) | - | 0 (0.00) |
| Denosumab | 0 (0.00) | 0 (0.00) | 0 (0.00) | - | 0 (0.00) |
| Raloxifene | 87 (3.61) | 74 (2.91) | 75 (3.03) | .327 | 236 (3.18) |
| ^a^The tertile values, in ppb, were as follows: T1: < 4.69; T2: >= 4.69 and < 7.76; T3: >= 7.76.  ^b^NO: nitrogen monoxide.  ^c^SD: standard deviation.  ^d^CCI score: Charlson Comorbidity Index score. | | | | | |
