# Supplementary material for: Association Between Long‑Term Exposure to Air Pollution and the Rate of Mortality After Hip Fracture Surgery in Patients Older Than 60 Years: Nationwide Cohort Study in Taiwan
Source: JMIR Public Health Surveill. 2024 Mar 18;10:e46591. doi: 10.2196/46591 (PMC10985614; doi:10.2196/46591)
Supplement: Multimedia Appendix 10 [file publichealth_v10i1e46591_app10.docx]

## Multimedia Appendix 10. Characteristics of the study population across the tertiles of NO_2_ exposure.

| **Characteristics** | **Tertiles^a^ of average daily NO_2_^b^, n (%)** | | | ***P* value** | **Total (N = 7426)** |
| --- | --- | --- | --- | --- | --- |
|  | **T1 (lowest) (n = 2475)** | **T2 (n = 2475)** | **T3 (highest) (n = 2476)** |  |  |
| **Death** | 197 (7.96) | 311 (12.57) | 421 (17.00) | <.001 | 929 (12.51) |
| **Men** | 889 (35.92) | 958 (38.71) | 1079 (43.58) | <.001 | 2926 (39.40) |
| **Age (years)** | | | | <.001 |  |
| 60 to 79 | 1345 (54.34) | 1323 (53.45) | 1198 (48.38) |  | 3866 (52.06) |
| ≥80 | 1130 (45.66) | 1152 (46.55) | 1278 (51.62) |  | 3560 (47.94) |
| Mean ± SD^c^ | 78.20 ± 8.04 | 78.28 ± 8.07 | 79.14 ± 8.08 | <.001 | 78.54 ± 8.07 |
| **Urbanization level** | | | | <.001 |  |
| 1 (highest) | 917 (37.05) | 1082 (43.72) | 1273 (51.41) |  | 3272 (44.06) |
| 2 | 1090 (44.04) | 897 (36.24) | 778 (31.42) |  | 2765 (37.23) |
| 3 | 297 (12.00) | 250 (10.10) | 164 (6.62) |  | 711 (9.57) |
| 4 (lowest) | 2 (.08) | 46 (1.86) | 64 (2.58) |  | 112 (1.51) |
| Unknown | 169 (6.83) | 200 (8.08) | 197 (7.96) |  | 566 (7.62) |
| **Insurance amount (New Taiwan Dollar)** | | | | <.001 |  |
| Financially dependent | 8 (.32) | 9 (.36) | 7 (.28) |  | 24 (.32) |
| 1 to 19 999 | 797 (32.20) | 1248 (50.42) | 1492 (60.26) |  | 3537 (47.63) |
| 20 000 to 39 999 | 1394 (56.32) | 683 (27.60) | 296 (11.95) |  | 2373 (31.96) |
| ≥40 000 | 32 (1.29) | 41 (1.66) | 46 (1.86) |  | 119 (1.60) |
| Unknown | 244 (9.86) | 494 (19.96) | 635 (25.65) |  | 1373 (18.49) |
| **CCI^d^ score (mean ± SD^c^)** | 4.36 ± 2.87 | 4.66 ± 3.01 | 4.69 ± 3.02 | <.001 | 4.57 ± 2.97 |
| **Hip fracture procedure** | | | | .038 |  |
| Closed reduction of fracture with internal fixation | 131 (5.29) | 143 (5.78) | 174 (7.03) |  | 448 (6.03) |
| Open reduction of fracture with internal fixation | 1359 (54.91) | 1323 (53.45) | 1275 (51.49) |  | 3957 (53.29) |
| Partial hip replacement | 985 (39.80) | 1009 (40.77) | 1027 (41.48) |  | 3021 (40.68) |
| **Co-medications** | 2108 (85.17) | 2149 (86.83) | 2087 (84.29) | .037 | 6344 (85.43) |
| **Anti-osteoporosis medication** | | | |  |  |
| Alendronate | 290 (11.72) | 239 (9.66) | 223 (9.01) | .004 | 752 (10.13) |
| Risedronate | 0 (0.00) | 0 (0.00) | 0 (0.00) | - | 0 (0.00) |
| Ibandronate | 4 (0.16) | 6 (0.24) | 1 (0.04) | .157 | 11 (0.15) |
| Zoledronic | 0 (0.00) | 0 (0.00) | 0 (0.00) | - | 0 (0.00) |
| Denosumab | 0 (0.00) | 0 (0.00) | 0 (0.00) | - | 0 (0.00) |
| Raloxifene | 78 (3.15) | 88 (3.56) | 70 (2.83) | .343 | 236 (3.18) |
| ^a^The tertile values, in ppb, were as follows: T1: < 15.87; T2: >= 15.87 and < 20.29; T3: >= 20.29.  ^b^NO_2_: nitrogen dioxide.  ^c^SD: standard deviation.  ^d^CCI score: Charlson Comorbidity Index score. | | | | | |
