# Supplementary material for: Association Between Long‑Term Exposure to Air Pollution and the Rate of Mortality After Hip Fracture Surgery in Patients Older Than 60 Years: Nationwide Cohort Study in Taiwan
Source: JMIR Public Health Surveill. 2024 Mar 18;10:e46591. doi: 10.2196/46591 (PMC10985614; doi:10.2196/46591)
Supplement: Multimedia Appendix 11 [file publichealth_v10i1e46591_app11.docx]

## Multimedia Appendix 11. Characteristics of the study population across the tertiles of THC exposure.

| **Characteristics** | **Tertiles^a^ of average daily THC^b^, n (%)** | | | ***P* value** | **Total (N = 7325)** |
| --- | --- | --- | --- | --- | --- |
|  | **T1 (lowest) (n = 2442)** | **T2 (n = 2441)** | **T3 (highest) (n = 2442)** |  |  |
| **Death** | 118 (4.83) | 231 (9.46) | 573 (23.46) | <.001 | 922 (12.59) |
| **Men** | 875 (35.83) | 951 (38.96) | 1068 (43.73) | <.001 | 2894 (39.51) |
| **Age (years)** | | | | <.001 |  |
| 60 to 79 | 1425 (58.35) | 1269 (51.99) | 1120 (45.86) |  | 3814 (52.07) |
| ≥80 | 1017 (41.65) | 1172 (48.01) | 1322 (54.14) |  | 3511 (47.93) |
| Mean ± SD^c^ | 77.24 ± 8.19 | 78.50 ± 8.21 | 79.85 ± 7.63 | <.001 | 78.53 ± 8.09 |
| **Urbanization level** | | | | <.001 |  |
| 1 (highest) | 1033 (42.30) | 1069 (43.79) | 1115 (45.66) |  | 3217 (43.92) |
| 2 | 1019 (41.73) | 913 (37.40) | 791 (32.39) |  | 2723 (37.17) |
| 3 | 231 (9.46) | 229 (9.38) | 251 (10.28) |  | 711 (9.71) |
| 4 (lowest) | 5 (.20) | 38 (1.56) | 69 (2.83) |  | 112 (1.53) |
| Unknown | 154 (6.31) | 192 (7.87) | 216 (8.85) |  | 562 (7.67) |
| **Insurance amount (New Taiwan Dollar)** | | | | <.001 |  |
| Financially dependent | 13 (.53) | 6 (.25) | 5 (.20) |  | 24 (.33) |
| 1 to 19 999 | 913 (37.39) | 1063 (43.55) | 1532 (62.74) |  | 3508 (47.89) |
| 20 000 to 39 999 | 1121 (45.91) | 838 (34.33) | 351 (14.37) |  | 2310 (31.54) |
| ≥40 000 | 49 (2.01) | 46 (1.88) | 24 (.98) |  | 119 (1.62) |
| Unknown | 346 (14.17) | 488 (19.99) | 530 (21.70) |  | 1364 (18.62) |
| **CCI^d^ score (mean ± SD^c^)** | 4.22 ± 2.90 | 4.60 ± 2.94 | 4.89 ± 3.03 | <.001 | 4.57 ± 2.97 |
| **Hip fracture procedure** | | | | .710 |  |
| Closed reduction of fracture with internal fixation | 145 (5.94) | 162 (6.64) | 140 (5.73) |  | 447 (6.10) |
| Open reduction of fracture with internal fixation | 1299 (53.19) | 1301 (53.30) | 1299 (53.19) |  | 3899 (53.23) |
| Partial hip replacement | 998 (40.87) | 978 (40.07) | 1003 (41.07) |  | 2979 (40.67) |
| **Co-medications** | 2068 (84.68) | 2095 (85.83) | 2094 (85.75) | .450 | 6257 (85.42) |
| **Anti-osteoporosis medication** | | | |  |  |
| Alendronate | 348 (14.25) | 225 (9.22) | 172 (7.04) | <.001 | 745 (10.17) |
| Risedronate | 0 (0.00) | 0 (0.00) | 0 (0.00) | - | 0 (0.00) |
| Ibandronate | 4 (0.16) | 7 (0.29) | 0 (0.00) | .013 | 11 (0.15) |
| Zoledronic | 0 (0.00) | 0 (0.00) | 0 (0.00) | - | 0 (0.00) |
| Denosumab | 0 (0.00) | 0 (0.00) | 0 (0.00) | - | 0 (0.00) |
| Raloxifene | 101 (4.14) | 70 (2.87) | 64 (2.62) | .006 | 235 (3.21) |
| ^a^The tertile values, in ppm, were as follows: T1: < 2.22; T2: >= 2.22 and < 2.36; T3: >= 2.36.  ^b^THC: total hydrocarbons.  ^c^SD: standard deviation.  ^d^CCI score: Charlson Comorbidity Index score. | | | | | |
