# Supplementary material for: Association Between Long‑Term Exposure to Air Pollution and the Rate of Mortality After Hip Fracture Surgery in Patients Older Than 60 Years: Nationwide Cohort Study in Taiwan
Source: JMIR Public Health Surveill. 2024 Mar 18;10:e46591. doi: 10.2196/46591 (PMC10985614; doi:10.2196/46591)
Supplement: Multimedia Appendix 12 [file publichealth_v10i1e46591_app12.docx]

## Multimedia Appendix 12. Characteristics of the study population across the tertiles of NMHC exposure.

| **Characteristics** | **Tertiles^a^ of average daily NMHC^b^, n (%)** | | | ***P* value** | **Total (N = 7325)** |
| --- | --- | --- | --- | --- | --- |
|  | **T1 (lowest) (n = 2442)** | **T2 (n = 2441)** | **T3 (highest) (n = 2442)** |  |  |
| **Death** | 215 (8.80) | 305 (12.49) | 402 (16.46) | <.001 | 922 (12.59) |
| **Men** | 918 (37.59) | 933 (38.22) | 1043 (42.71) | <.001 | 2894 (39.51) |
| **Age (years)** | | | | <.001 |  |
| 60 to 79 | 1316 (53.89) | 1315 (53.87) | 1183 (48.44) |  | 3814 (52.07) |
| ≥80 | 1126 (46.11) | 1126 (46.13) | 1259 (51.56) |  | 3511 (47.93) |
| Mean ± SD^c^ | 78.28 ± 7.99 | 78.22 ± 8.24 | 79.09 ± 7.99 | <.001 | 78.53 ± 8.09 |
| **Urbanization level** | | | | <.001 |  |
| 1 (highest) | 965 (39.52) | 991 (40.60) | 1261 (51.64) |  | 3217 (43.92) |
| 2 | 1021 (41.81) | 953 (39.04) | 749 (30.67) |  | 2723 (37.17) |
| 3 | 323 (13.23) | 225 (9.22) | 163 (6.67) |  | 711 (9.71) |
| 4 (lowest) | 1 (.04) | 47 (1.93) | 64 (2.62) |  | 112 (1.53) |
| Unknown | 132 (5.41) | 225 (9.22) | 205 (8.39) |  | 562 (7.67) |
| **Insurance amount (New Taiwan Dollar)** | | | | <.001 |  |
| Financially dependent | 10 (.41) | 8 (.33) | 6 (.25) |  | 24 (.33) |
| 1 to 19 999 | 797 (32.64) | 1216 (49.82) | 1495 (61.22) |  | 3508 (47.89) |
| 20 000 to 39 999 | 1354 (55.45) | 662 (27.12) | 294 (12.04) |  | 2310 (31.54) |
| ≥40 000 | 32 (1.31) | 40 (1.64) | 47 (1.92) |  | 119 (1.62) |
| Unknown | 249 (10.20) | 515 (21.10) | 600 (24.57) |  | 1364 (18.62) |
| **CCI^d^ score (mean ± SD^c^)** | 4.43 ± 2.88 | 4.68 ± 2.98 | 4.61 ± 3.03 | .011 | 4.57 ± 2.97 |
| **Hip fracture procedure** | | | | .024 |  |
| Closed reduction of fracture with internal fixation | 134 (5.49) | 142 (5.82) | 171 (7.00) |  | 447 (6.10) |
| Open reduction of fracture with internal fixation | 1355 (55.49) | 1281 (52.48) | 1263 (51.72) |  | 3899 (53.23) |
| Partial hip replacement | 953 (39.03) | 1018 (41.70) | 1008 (41.28) |  | 2979 (40.67) |
| **Co-medications** | 2095 (85.79) | 2113 (86.56) | 2049 (83.91) | .026 | 6257 (85.42) |
| **Anti-osteoporosis medication** | | | |  |  |
| Alendronate | 301 (12.33) | 235 (9.63) | 209 (8.56) | <.001 | 745 (10.17) |
| Risedronate | 0 (0.00) | 0 (0.00) | 0 (0.00) | - | 0 (0.00) |
| Ibandronate | 6 (0.25) | 4 (0.16) | 1 (0.04) | .183 | 11 (0.15) |
| Zoledronic | 0 (0.00) | 0 (0.00) | 0 (0.00) | - | 0 (0.00) |
| Denosumab | 0 (0.00) | 0 (0.00) | 0 (0.00) | - | 0 (0.00) |
| Raloxifene | 86 (3.52) | 80 (3.28) | 69 (2.83) | .375 | 235 (3.21) |
| ^a^The tertile values, in ppm, were as follows: T1: < .25; T2: >= .25 and < .32; T3: >= .32.  ^b^NMHC: non-methane hydrocarbon.  ^c^SD: standard deviation.  ^d^CCI score: Charlson Comorbidity Index score. | | | | | |
