# Supplementary material for: Association Between Long‑Term Exposure to Air Pollution and the Rate of Mortality After Hip Fracture Surgery in Patients Older Than 60 Years: Nationwide Cohort Study in Taiwan
Source: JMIR Public Health Surveill. 2024 Mar 18;10:e46591. doi: 10.2196/46591 (PMC10985614; doi:10.2196/46591)
Supplement: Multimedia Appendix 13 [file publichealth_v10i1e46591_app13.docx]

## Multimedia Appendix 13. Characteristics of the study population across the tertiles of CH_4_ exposure.

| **Characteristics** | **Tertiles^a^ of average daily CH_4_^b^, n (%)** | | | ***P* value** | **Total (N = 7325)** |
| --- | --- | --- | --- | --- | --- |
|  | **T1 (lowest) (n = 2442)** | **T2 (n = 2441)** | **T3 (highest) (n = 2442)** |  |  |
| **Death** | 100 (4.10) | 229 (9.38) | 593 (24.28) | <.001 | 922 (12.59) |
| **Men** | 867 (35.50) | 995 (40.76) | 1032 (42.26) | <.001 | 2894 (39.51) |
| **Age (years)** | | | | <.001 |  |
| 60 to 79 | 1410 (57.74) | 1264 (51.78) | 1140 (46.68) |  | 3814 (52.07) |
| ≥80 | 1032 (42.26) | 1177 (48.22) | 1302 (53.32) |  | 3511 (47.93) |
| Mean ± SD^c^ | 77.23 ± 8.22 | 78.38 ± 8.19 | 79.98 ± 7.60 | <.001 | 78.53 ± 8.09 |
| **Urbanization level** | | | | <.001 |  |
| 1 (highest) | 1138 (46.60) | 1089 (44.61) | 990 (40.54) |  | 3217 (43.92) |
| 2 | 878 (35.95) | 907 (37.16) | 938 (38.41) |  | 2723 (37.17) |
| 3 | 229 (9.38) | 212 (8.68) | 270 (11.06) |  | 711 (9.71) |
| 4 (lowest) | 29 (1.19) | 51 (2.09) | 32 (1.31) |  | 112 (1.53) |
| Unknown | 168 (6.88) | 182 (7.46) | 212 (8.68) |  | 562 (7.67) |
| **Insurance amount (New Taiwan Dollar)** | | | | <.001 |  |
| Financially dependent | 12 (.49) | 8 (.33) | 4 (.16) |  | 24 (.33) |
| 1 to 19 999 | 1075 (44.02) | 1105 (45.27) | 1328 (54.38) |  | 3508 (47.89) |
| 20 000 to 39 999 | 875 (35.83) | 799 (32.73) | 636 (26.04) |  | 2310 (31.54) |
| ≥40 000 | 53 (2.17) | 48 (1.97) | 18 (.74) |  | 119 (1.62) |
| Unknown | 427 (17.49) | 481 (19.71) | 456 (18.67) |  | 1364 (18.62) |
| **CCI^d^ score (mean ± SD^c^)** | 4.20 ± 2.91 | 4.61 ± 2.94 | 4.91 ± 3.01 | <.001 | 4.57 ± 2.97 |
| **Hip fracture procedure** | | | | .418 |  |
| Closed reduction of fracture with internal fixation | 161 (6.59) | 149 (6.10) | 137 (5.61) |  | 447 (6.10) |
| Open reduction of fracture with internal fixation | 1274 (52.17) | 1293 (52.97) | 1332 (54.55) |  | 3899 (53.23) |
| Partial hip replacement | 1007 (41.24) | 999 (40.93) | 973 (39.84) |  | 2979 (40.67) |
| **Co-medications** | 2058 (84.28) | 2108 (86.36) | 2091 (85.63) | .112 | 6257 (85.42) |
| **Anti-osteoporosis medication** | | | |  |  |
| Alendronate | 329 (13.47) | 253 (10.36) | 163 (6.67) | <.001 | 745 (10.17) |
| Risedronate | 0 (0.00) | 0 (0.00) | 0 (0.00) | - | 0 (0.00) |
| Ibandronate | 4 (0.16) | 5 (0.20) | 2 (0.08) | .491 | 11 (0.15) |
| Zoledronic | 0 (0.00) | 0 (0.00) | 0 (0.00) | - | 0 (0.00) |
| Denosumab | 0 (0.00) | 0 (0.00) | 0 (0.00) | - | 0 (0.00) |
| Raloxifene | 99 (4.05) | 71 (2.91) | 65 (2.66) | .013 | 235 (3.21) |
| ^a^The tertile values, in ppm, were as follows: T1: < 1.96; T2: >= 1.96 and < 2.05; T3: >= 2.05.  ^b^CH_4_: methane.  ^c^SD: standard deviation.  ^d^CCI score: Charlson Comorbidity Index score. | | | | | |
