# Supplementary material for: Association Between Long‑Term Exposure to Air Pollution and the Rate of Mortality After Hip Fracture Surgery in Patients Older Than 60 Years: Nationwide Cohort Study in Taiwan
Source: JMIR Public Health Surveill. 2024 Mar 18;10:e46591. doi: 10.2196/46591 (PMC10985614; doi:10.2196/46591)
Supplement: Multimedia Appendix 14 [file publichealth_v10i1e46591_app14.docx]

## Multimedia Appendix 14. Pearson’s correlation analysis for air pollutants detected over the exposure period.

|  | **SO_2_^a^** | **CO_2_^b^** | **CO^c^** | **O_3_^d^** | **PM_10_^e^** | **PM_2.5_^f^** | **NO_X_^g^** | **NO^h^** | **NO_2_^i^** | **THC^j^** | **NMHC^k^** | **CH_4_^l^** |
| --- | --- | --- | --- | --- | --- | --- | --- | --- | --- | --- | --- | --- |
| **SO_2_^a^** | 1 | .005 | .264*** | -.001 | .589*** | .601*** | .360*** | .182*** | .513*** | .201*** | .221*** | .099*** |
| **CO_2_^b^** |  | 1 | -.324*** | .150*** | .314*** | .171*** | -.194*** | -.251*** | -.116*** | -.443*** | -.390*** | -.344*** |
| **CO^c^** |  |  | 1 | -.616*** | -.180*** | -.082*** | .949*** | .928*** | .879*** | .747*** | .906*** | .288*** |
| **O_3_^d^** |  |  |  | 1 | .244*** | .200*** | -.525*** | -.463*** | -.539*** | -.470*** | -.446*** | -.304*** |
| **PM_10_^e^** |  |  |  |  | 1 | .911*** | -.137*** | -.315*** | .064*** | -.048*** | -.251*** | .174*** |
| **PM_2.5_^f^** |  |  |  |  |  | 1 | -.065*** | -.246*** | .135*** | -.037** | -.187*** | .130*** |
| **NO_X_^g^** |  |  |  |  |  |  | 1 | .956*** | .950*** | .693*** | .890*** | .218*** |
| **NO^h^** |  |  |  |  |  |  |  | 1 | .816*** | .731*** | .924*** | .244*** |
| **NO_2_^i^** |  |  |  |  |  |  |  |  | 1 | .586*** | .767*** | .168*** |
| **THC^j^** |  |  |  |  |  |  |  |  |  | 1 | .802*** | .799*** |
| **NMHC^k^** |  |  |  |  |  |  |  |  |  |  | 1 | .282*** |
| **CH_4_^l^** |  |  |  |  |  |  |  |  |  |  |  | 1 |
| ^a^SO_2_: sulfur dioxide.  ^b^CO_2_: carbon dioxide.  ^c^CO: carbon monoxide.  ^d^O_3_: ozone.  ^e^PM_10_: particulate matters having a size of <10 μm.  ^f^PM_2.5_: particulate matters having a size of <2.5 μm.  ^g^NO_X_: nitrogen oxides.  ^h^NO: nitrogen monoxide.  ^i^NO_2_: nitrogen dioxide.  ^j^THC: total hydrocarbons.  ^k^NMHC: nonmethane hydrocarbons.  ^l^CH_4_: methane.  ***Correlation significant at the .001 level (two-tailed).  **Correlation significant at the .01 level (two-tailed).  Absolute value of correlation coefficient values of <.3 denote a low strength of correlation, which qualified as the controlling pollutant in multiple-pollutant models of targeted pollutants. | | | | | | | | | | | | |
