# Supplementary material for: Association Between Long‑Term Exposure to Air Pollution and the Rate of Mortality After Hip Fracture Surgery in Patients Older Than 60 Years: Nationwide Cohort Study in Taiwan
Source: JMIR Public Health Surveill. 2024 Mar 18;10:e46591. doi: 10.2196/46591 (PMC10985614; doi:10.2196/46591)
Supplement: Multimedia Appendix 15 [file publichealth_v10i1e46591_app15.docx]

## Multimedia Appendix 15. Hazard ratios for long-term ozone exposure at 1 standard deviation increment associated with mortality rate.

| **Controlling pollutants** | **Adjusted^a^ HR^b^ (95% CI^c^)** | ***P* values** |
| --- | --- | --- |
| - | 0.65 (0.60,0.69) | <.001 |
| CO_2_^d^ | 0.77 (0.65,0.91) | .002 |
| PM_10_^e^ | 0.61 (0.56,0.66) | <.001 |
| PM_2.5_^f^ | 0.63 (0.57,0.68) | <.001 |
| CO_2_^d^, PM_2.5_^f^ | 0.82 (0.69,0.98) | .033 |
| ^a^Cox regression models were adjusted for age, urbanization level, insurance amount, CCI score, hip fracture procedure, co-medications, anti-osteoporosis medication, ambient temperature, season, lag0−1 and controlled pollutants^g^ (weak correlation with O_3_).  ^b^HR: hazard ratio.  ^c^CI: confidence interval.  ^d^CO_2_: carbon dioxide.  ^e^PM_10_: particulate matters having a size of <10 μm.  ^f^PM_2.5_: particulate matters having a size of <2.5 μm.  ^g^Additional pollutants were added into the pollutant models for multiple analysis only when Pearson’s correlation coefficient was <0.3. | | |
